# Electrolytic Synthesis of White Phosphorus Is Promoted in Oxide-Deficient Molten Salts

**DOI:** 10.1021/acscentsci.2c01336

**Published:** 2023-02-21

**Authors:** Jonathan F. Melville, Andrew J. Licini, Yogesh Surendranath

**Affiliations:** Department of Chemistry, Massachusetts Institute of Technology, Cambridge, Massachusetts02139, United States

## Abstract

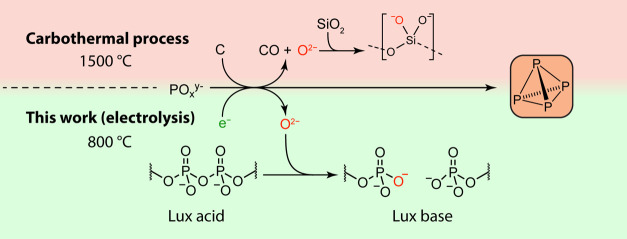

Elemental white phosphorus (P_4_) is a key feedstock for the entire phosphorus-derived chemicals industry, spanning everything from herbicides to food additives. The electrochemical reduction of phosphate salts could enable the sustainable production of P_4_; however, such electrosynthesis requires the cleavage of strong, inert P–O bonds. By analogy to the promotion of bond activation in aqueous electrolytes with high proton activity (Brønsted–Lowry acidity), we show that low oxide anion activity (Lux–Flood acidity) enhances P–O bond activation in molten salt electrolytes. We develop electroanalytical tools to quantify the oxide dependence of phosphate reduction, and find that Lux acidic phosphoryl anhydride linkages enable selective, high-efficiency electrosynthesis of P_4_ at a yield of 95% Faradaic efficiency. These fundamental studies provide a foundation that may enable the development of low-carbon alternatives to legacy carbothermal synthesis of P_4_.

## Abstract 2

Carbothermal production of white phosphorus underpins the food, herbicide, battery, and computer industries. Molten polyphosphate electrolysis is presented as a greener, highly selective alternative.

## Introduction

**Figure 1:**
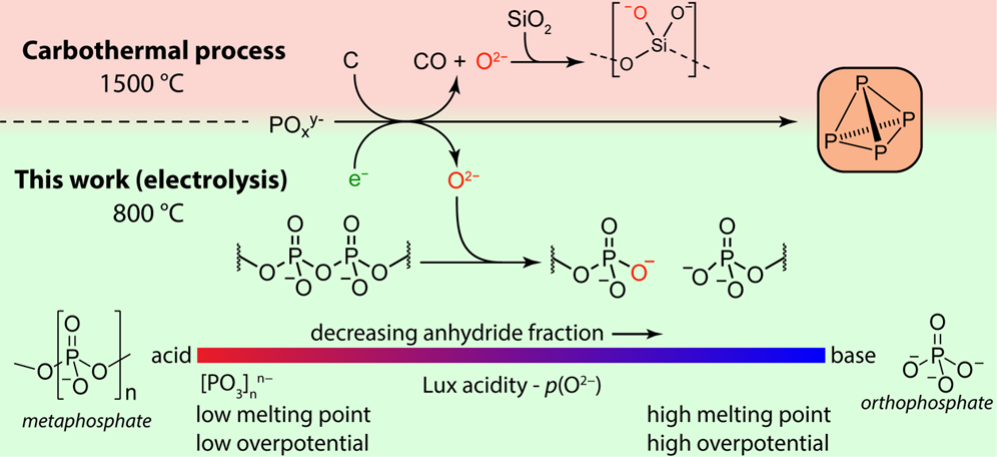
When white phosphorus, P_4_, is prepared by the incumbent carbothermal process (top), generated oxide equivalents are accepted by SiO_2_ to produce metasilicate slag. In the presented electrosynthetic route to P_4_ (bottom), oxide equivalents are instead absorbed by cleavage of phosphoryl anhydride linkages natively present in the Lux acidic condensed phosphate melt.

In the natural world, phosphorus exists predominantly in the +5 oxidation state as phosphate.^1^ Despite this, the element exhibits a rich variety of oxidation states and corresponding reactivity, making it a critical component to a plethora of chemical reagents, materials, and intermediates. Both lower-valent phosphorus fine chemicals and pentavalent phosphorus commodity chemicals—from the ubiquitous herbicide glyphosate to the food-grade phosphoric acid in a can of soda—are produced via the intermediacy of P^(0)^ in elemental white phosphorus, P_4_.^2,3^ This roundabout reduction–reoxidation sequence is employed in part to overcome the incredible stability of the phosphate P–O bond (595 kJ/mol),^4^ which resists facile substitution or elimination chemistries that would enable the direct conversion of raw phosphate to valuable phosphites or phosphonates. Despite emerging alternative processes,^5,6^ the phosphorus chemical value chain relies upon the “thermal process” (Figure 1, top; eq 1), which employs extremely forcing conditions to drive the reduction of phosphate to P_4_ by carbon coke.
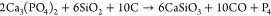
1

The anatomy of this reaction reflects the strength of the P–O bond. An arc furnace is used to achieve extreme temperatures up to 1500 °C, necessary both to sufficiently soften the reactants and to provide an entropic driving force—in the process limiting the coke to incomplete oxidation to CO.^7^ The role of SiO_2_ is less obvious but no less crucial: it absorbs the oxide ions that (by basic laws of atom and charge conservation) must necessarily be generated when anionic PO_4_^3–^ is reduced to neutral P_4_. This process may be understood through the lens of Lux–Flood acid–base theory, with orthophosphate acting as a “Lux basic” oxide donor and silica acting as a “Lux acidic” oxide acceptor.^8,9^ This oxide transfer produces a molten calcium metasilicate slag, the heat content of which carries away 30% of the energy input of the overall process.^1,10,11^ The emerging imperative to green this difficult-to-decarbonize process exposes a fundamental scientific challenge: the efficient activation of P–O bonds using renewable energy inputs.

Akin to the Hall process for aluminum synthesis, the net 20-electron reduction of phosphates to phosphorus (henceforth the phosphate reduction reaction, PRR) can, in principle, be driven electrochemically. However, numerous additional constraints must be met to enable efficient PRR: Given the low reduction potential and kinetic complexity of PRR, the electrolyte must be designed to inhibit parasitic and kinetically facile hydrogen evolution. The system should allow for efficient product separation from the reaction media. The system should inhibit the formation of electrically-insulating red phosphorus deposits that could passivate the electrode. Any viable system for electrochemical conversion of phosphates to phosphorus must contend with these hurdles, over and above the formidable challenge of activating inert P–O bonds in the first place.

The electrolysis of molten salts addresses these obstacles. The natively high ionic strength of salt melts enables high conductivities, while the low proton content precludes H_2_ evolution. By operating at temperatures above 281 °C,^12^ such systems avoid the production of passivating red phosphorus in favor of gaseous white phosphorus, which may be driven out of the reactor headspace. Due to the intrinsic separation of cathodic and anodic half-reactions in an electrochemical cell, this approach allows for the segregation of the highly oxophilic P_4_ generated at the cathode from the oxygen evolved at the anode.

**Scheme 1:**
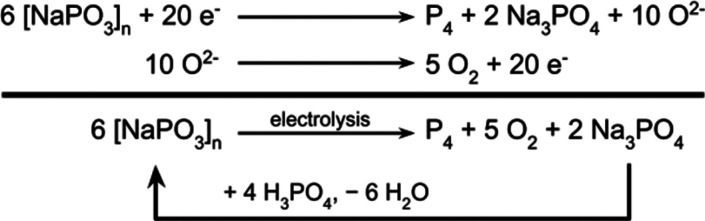
Half-Reaction and Overall Electrolysis Scheme for Metaphosphate Electrolysis and Regeneration

We chose to investigate molten condensed phosphate salts, which consist of phosphate oligomers bridged by phosphoryl anhydride linkages (Figure S18). In addition to their high intrinsic phosphate content—which ensures high diffusion-limited current densities—we theorized that these phosphoryl anhydride linkages would serve as Lux acids analogous to the oxide-accepting SiO_2_ in the thermal process, activating the strong P–O bonds and thereby promoting their reduction to P_4_ (Figure 1, bottom; Figure S19). The addition of phosphoryl anhydride linkages also dramatically lowers the fusion temperature of these salt melts, from 1583 °C for sodium orthophosphate, Na_3_PO_4_,^12^ to a mere 628 °C for sodium metaphosphate, [NaPO_3_]_*n*_.^13^ As written in Scheme 1, the putative electrolysis of sodium metaphosphate would, in principle, generate P_4_, O_2_, and Na_3_PO_4_. Although there is no appreciable concentration of free oxide ions in condensed phosphate melts, the half reactions in Scheme 1 are written in a general form with reference to “O^2–^” to align with the formal definition of Lux acids and bases as oxide acceptor and donors, respectively. This is analogous to the common notation of aqueous electrochemical half reactions with reference to free “H^+^” despite the strong solvation of protons in aqueous media. The Na_3_PO_4_ produced in the overall reaction dissolves into the melt by cleaving phosphoryl anhydride linkages, thereby decreasing the Lux acidity of the bulk electrolyte. These linkages may be regenerated by the addition and subsequent thermal dehydration of phosphoric acid, restoring the Lux acidity of the melt and (in net) effecting the electrolysis of phosphoric acid to white phosphorus, oxygen, and water (eq 2).
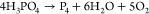
2

Historically, the electrolysis of molten metaphosphates has been investigated in the context of a prospective flux for metal electrodeposition,^14−23^ although research in this area was largely abandoned in the early 1970s. While the electroreduction of metaphosphates was observed with concomitant evolution of P_4_, these studies provide few quantitative insights or synthetic handles for modulating the efficiency or selectivity of phosphate reduction, limiting the development of practical phosphorus electrosynthesis technologies. Owing to the complexities of precise electroanalysis in this exotic reaction medium—and, presumably, minimal contemporaneous environmental incentive to decarbonize the incumbent thermal process—these knowledge gaps persist to the present day. A resurgent interest in low-carbon P_4_ synthesis has brought forth a recent report in which orthophosphates are electrolyzed in a calcium chloride melt;^24^ however, this system is limited by a low orthophosphate solubility in the reaction medium (3 mass % at 800 °C), the possibility of undesired chloride oxidation and subsequent chlorocarbon formation at the anode,^25,26^ and a lack of oxide-transporting moieties to mediate stoichiometric balance with the anodic reaction.

By comparison, condensed phosphates comprise not an isolated set of reaction conditions but a broad reaction space, enabling transverse studies across a continuum of electrolytes to directly interrogate the role of Lux acidic moieties in promoting electrochemical P–O activation. This complexity poses its own challenges, as applying principles of rational design to this multifaceted electrolyte canvas necessitates the development of heretofore-nonexistent electroanalytical methodologies to enable objective comparison across a series of melt compositions. To this end, we present a toolbox of techniques for quantifying reaction overpotential, selectivity, and efficiency across a range of electrolyte compositions, and employ these tools to elucidate the governing attributes which underpin the promotion of P_4_ electrosynthesis by phosphoryl anhydride linkages.

## Results and Discussion

**Figure 2:**
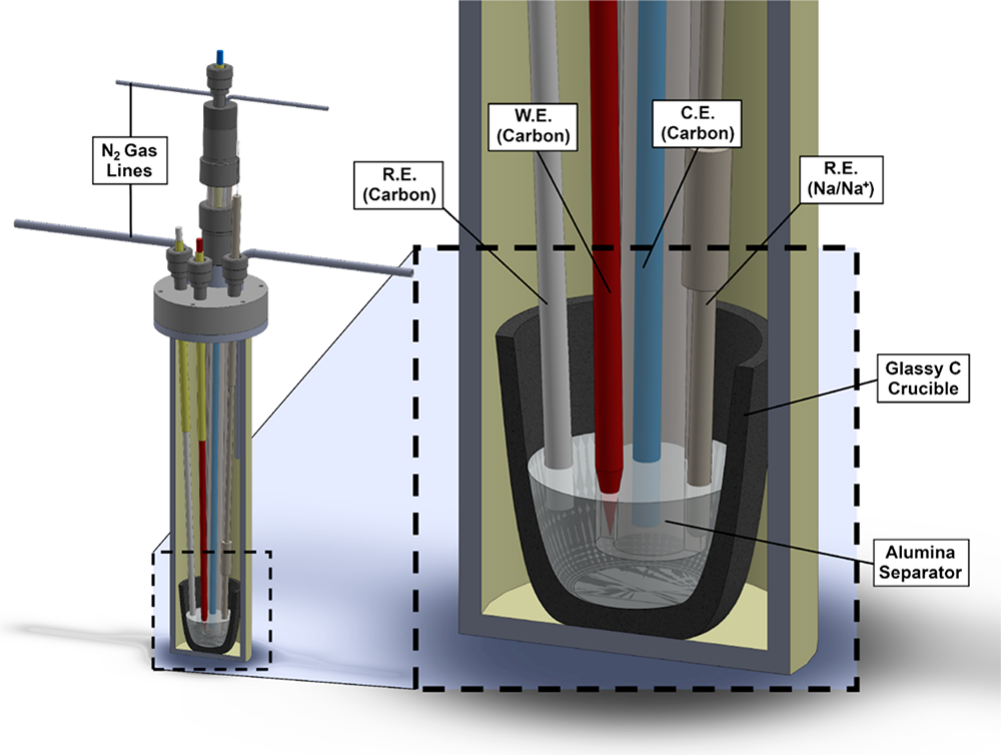
Cutaway schematic of a high-temperature electrochemical cell featuring separated working and counter chambers and a Na/Na^+^ reference electrode. W.E. = working electrode; R.E. = reference electrode; C.E. = counter electrode.

We began by constructing a robust system for quantitative high-temperature electroanalysis and mechanistic investigation of molten salt electrolysis (Figure 2 and Figures S1–S4). Our cell included separated anodic and cathodic chambers with independent N_2_ flow streams, a Na/Na^+^ electrode for use as a fixed-potential reference, and a cold trap for product characterization and quantification. Graphite rods were used for all electrodes in a standard 3-electrode analytical setup (working, counter, and reference). Whereas many transition metals readily form metal phosphide alloys,^27,28^ carbon is highly immiscible with phosphorus;^29,30^ accordingly, we found graphite to be stable under cathodic conditions, displaying no significant changes in mass nor surface area under any conditions examined in this study. By comparison, the β-alumina membrane of the Na/Na^+^ reference electrode was subject to corrosion upon prolonged exposure to the melt; as a result, it was instead used to calibrate a graphite pseudoreference electrode, the two comprising a quasireference electrode pair (Figure S5).^31^ Since these studies focus on the promotion of the cathodic half-reaction, our setup employed a sacrificial graphite counter electrode which underwent oxidation principally to CO_2_, as determined by GC analysis (see Section S1.6 for additional details). Robust experimental design is essential for reproducible electrochemistry at high temperatures, and as such we provide in our Supporting Information full specifications and part numbers for construction of the molten salt electrolysis system employed in these studies.

**Figure 3:**
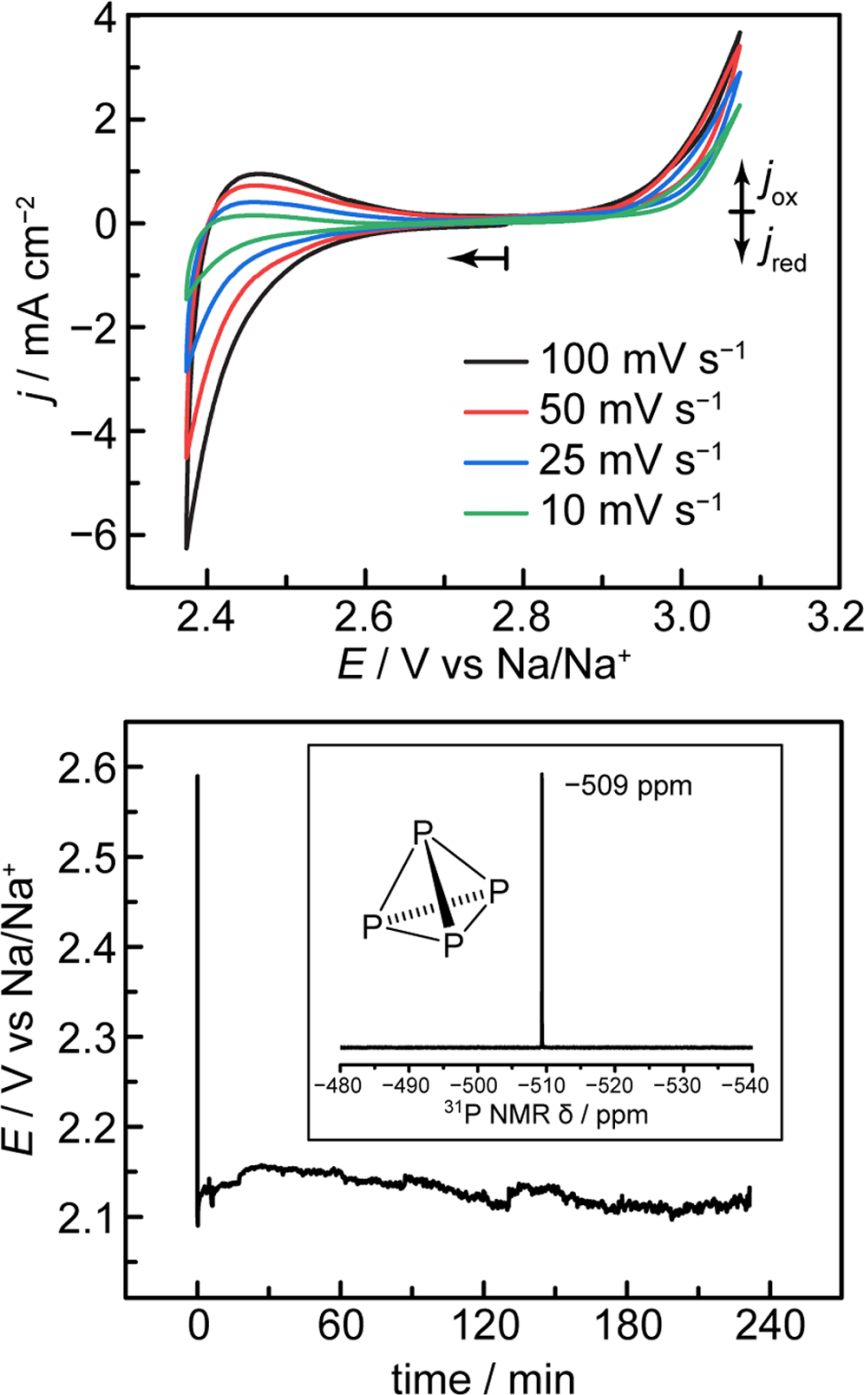
(top) Cyclic voltammograms of a graphite electrode in molten sodium trimetaphosphate at 800 °C. Arrow indicates point of initiation and direction of scan. (bottom) Chronopotentiometry trace of molten sodium trimetaphosphate electrolysis at a current density of 48 mA cm^–2^. (bottom, inset) Proton-coupled ^31^P NMR spectrum of electrogenerated P_4_ dissolved in CS_2_.

Using this molten salt electroanalytical setup, we investigated the electrochemical behavior of molten sodium trimetaphosphate using cyclic voltammetry (CV), as seen in Figure 3. To mitigate the effects of bubble formation and associated occlusion of the electrode surface area, we employed a graphite working electrode with a conical tip (Figure 2, red; Figure S4b). Voltammetric data reveal the onset of substantial reductive current at approximately +2.4 V against a Na/Na^+^ reference electrode. As our electrolyte is itself our reactant, this cathodic wave does not peak; rather, we observe an exponentially rising reductive feature of the type associated with solvent reduction in conventional electrolytes. However, unlike a typical catalytic wave, this cathodic process displays a square-root dependence on scan rate, implying the participation of a diffusion-limited species. Upon reversal of the potential sweep, we observe the immediate appearance of a broad oxidative wave, which we ascribe to a reverse redox process. The peak potential and current magnitude in this anodic wave are also scan-rate-dependent; a prominent oxidative feature is observed at 100 mV s^–1^, whereas it is almost nonexistent at 10 mV s^–1^. These data are characteristic of the formation and build-up of a transient reduced species at the electrode interface. To further probe the reductive voltammetry, we performed computational CV simulations (Tables S1–S3 and Figures S7 and S8), which indicated that the voltammetric shape and scan rate dependence could result from diffusion-limited back-oxidation of partially reduced intermediates in a reversible redox process. Finally, at +3.0 V vs Na/Na^+^ we observe an irreversible anodic feature with minimal scan rate dependence, which we assign to the oxidation of our graphite electrode; prolonged electrolysis at this potential will result in visible corrosion of the otherwise-inert graphite.

Steady-state electrolysis was used to determine the product of electroreduction and the Faradaic efficiency of its formation. Bulk electrolysis at a fixed current density of 48 mA cm^–2^ results in a steady potential of +2.1 V vs Na/Na^+^ (Figure 3, bottom) over a time scale of hours (see Section S2.4 for additional details). Electrolysis in this potential region with sustained flow of N_2_ gas through the cathode chamber results in the formation of pyrophoric yellow-white crystals (Figure S9a) in the cold trap. ^31^P NMR analysis of these waxy crystals in carbon disulfide (Figure 3b, bottom inset) reveals a single peak at a chemical shift of −509.4 ppm vs an 85% H_3_PO_4_ reference, which is highly characteristic of white phosphorus.^32^ If the glass trap is not lined with aluminum foil, these crystals will slowly decay over the course of several hours into a reddish-brown coating (Figure S9b), consistent with the photoconversion of molecular white phosphorus to polymeric red phosphorus. Additional accumulations of red phosphorus accrete on tubing and surfaces near the reactor head (Figure S10). These products are generated exclusively upon the passage of current, as collection experiments performed at open circuit over the course of 12 h yielded no condensate on the reactor head nor observable products in the bleach or cold traps. Following electrolysis, red and white phosphorus residues in the trap and associated transfer lines were oxidized with bleach for quantification by integration of proton-coupled ^31^P NMR against an internal standard (see Section S1.4 and Figures S11 and S12). While the isolated yield of volatilized phosphorus products is highly dependent on the integrity of the O-ring reactor seals at temperature, we observe under optimal conditions a peak isolated yield of elemental phosphorus of 64.5% relative to the charge passed, for the 4 h galvanostatic electrolysis pictured in Figure 3. Accounting for the 68% reactor gas-flow retention rate (thereby accounting for P_4_ losses from gas leakage), this isolated yield corresponds to a Faradaic yield of 95% for P_4_ evolution. We note that this gas leakage is attributed to the poor thermal stability of the perfluoroelastomer O-ring seals employed in our laboratory-scale electrochemical setup; incumbent electrochemical devices (such as solid-oxide electrolyzers) utilize metal-, ceramic-, and/or glass-based seals that are stable to higher temperatures and pressures than those demanded in this process,^33^ and, thus, we envision that increased gas collection efficiency can be realized with improved engineering of a larger-scale reactor. In line with this high estimated FE for P_4_ production, postelectrolysis analysis of the electrode surface by XPS and the electrolyte by ^31^P NMR shows no accumulation of P(III) species (see Sections S1.4b and S2.6). In contrast to legacy and contemporaneous studies of phosphate electrolysis that omit quantitative product analysis,^14−24^ our spectroscopic and Faradaic efficiency data provide the first numerical evidence that P_4_ is the predominant cathodic product of metaphosphate electrolysis.

As there exists a paucity of thermochemical reference data on condensed phosphate melts, a theoretical energetic efficiency for this process cannot be readily calculated, and must instead be measured empirically. In particular, we must establish the excess energy or overpotential, η, required to drive PRR in this system relative to the thermodynamic minimum. Calculating η requires knowledge of the thermodynamic equilibrium reduction potential *E*_eq_ for the P^V^ to P^(0)^ redox couple under the conditions of our electrolysis, as the former is defined in terms of the latter: *E* = *E*_eq_ + η. Insight into the equilibrium potential of the reaction requires a direct probe of this minimum potential at which the reverse oxidation of P^(0)^ to P^V^ can occur; however, our cathodic product, white phosphorus, rapidly escapes from the electrode interface due to its gaseous nature at the temperature of reaction. To increase the residency time of the P^(0)^ product, we employed a graphite cathode with a concave depression capable of trapping a P_4_ bubble (Figure S4c), with the aim of establishing a PO_3_^–^ ⇌ P_4_ equilibrium at the graphite–metaphosphate–phosphorus triple-phase boundary. For an electrode that can catalyze both anodic and cathodic half-reactions, the open-circuit potential (OCP) recorded in the presence of reactant, polyphosphate, and the product, white phosphorus gas, provides an estimate of the equilibrium potential *E*_eq_ for the reaction.^34^

**Figure 4:**
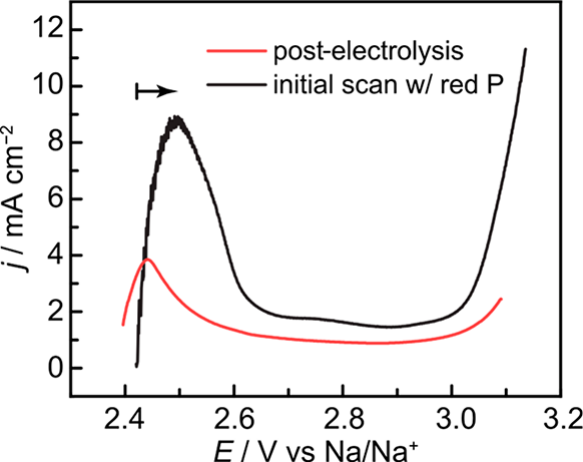
Linear-sweep voltammograms scanning positively from OCP at 100 mV s^–1^ with hollowed graphite electrodes containing electrosynthesized (red) and chemically generated (black) P_4_.

**Figure 5:**
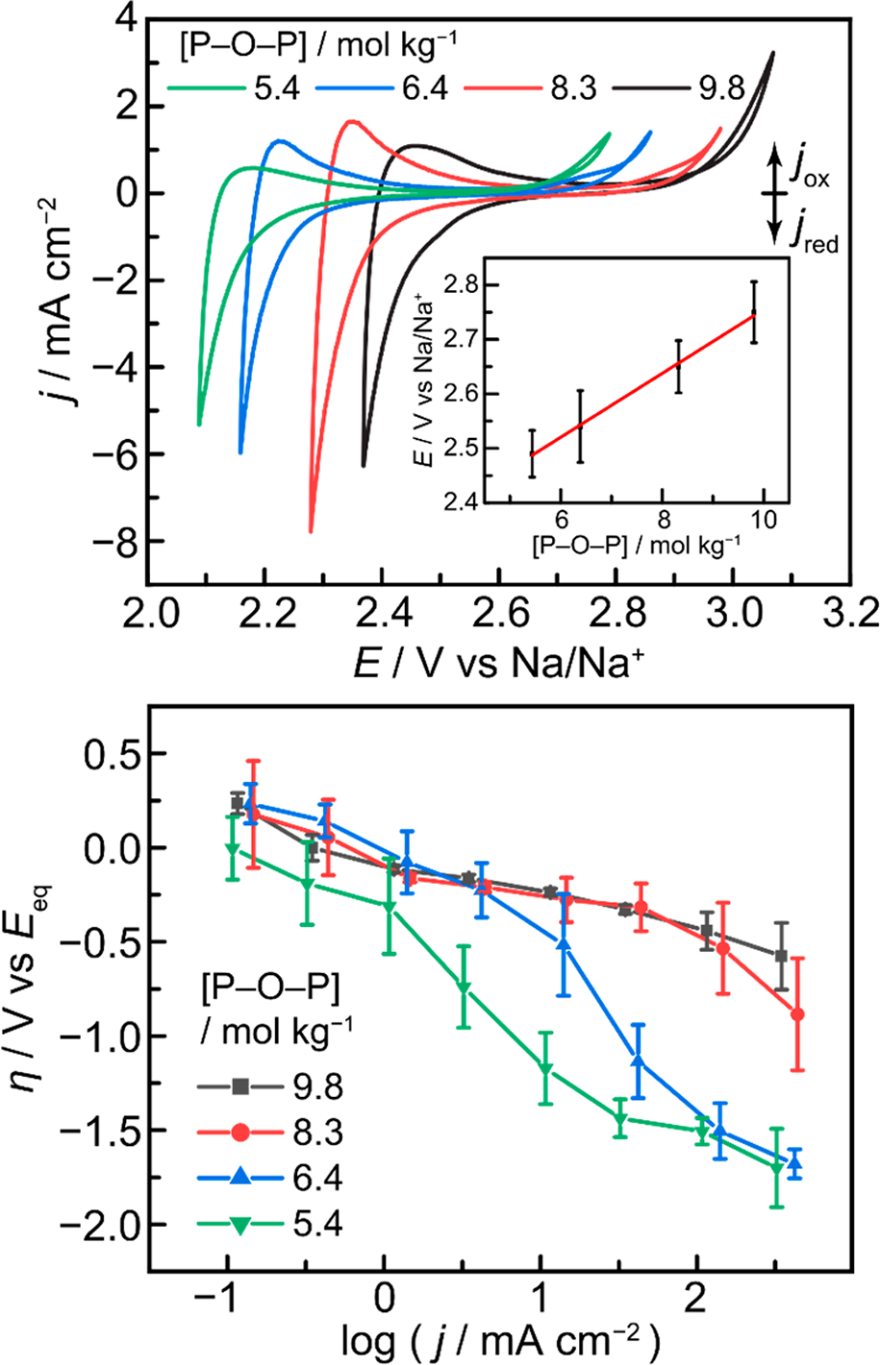
(top) Cyclic voltammograms (scan rate = 100 mV s^–1^) of condensed sodium phosphate melts. [P–O–P] denotes the molal concentration of phosphoryl anhydride linkages in the melt, corresponding in decreasing order to the anhydride concentrations of pure sodium metaphosphate, decapolyphosphate, tetrapolyphosphate, and tripolyphosphate melts. (top, inset) Plot of graphite pseudoreference open-circuit potentials against a Na/Na^+^ reference electrode as a function of phosphoryl anhydride linkage concentration. Error bars represent 95% confidence intervals as collected from three independent experimental setups. (bottom) Plot of phosphate reduction overpotential as a function of chronopotentiometric current density in sodium trimetaphosphate, decapolyphosphate, tetrapolyphosphate, and tripolyphosphate melts. Error bars represent 95% confidence intervals of a set of three independent experimental setups, each setup itself being performed in triplicate, for a total of nine data points.

We implemented this approach for measuring the PRR equilibrium potential, *E*_eq_, by galvanostatically generating a P_4_ bubble in the hollow of our working electrode and performing a linear-sweep voltammogram scanning positively from the OCP (Figure 4, red trace). We observe an anodic peak at approximately +2.45 V vs Na/Na^+^, paralleling that seen in our cyclic voltammogram and with similar scan rate dependence (Figure S15). This indicates that our concave electrode is capable of trapping comparatively long-lived partially reduced species at the interface, as well as subsequently reoxidizing these species. While highly suggestive that the voltammetric feature at +2.45 V vs Na/Na^+^ corresponds to P_4_ oxidation, this evidence does not by itself confirm that white phosphorus (or a species in a rapid chemical equilibrium with white phosphorus) is the source of anodic current. To further probe the nature of the oxidative wave, we conducted a product dosing experiment by exploiting the thermal depolymerization of red phosphorus to white phosphorus. We loaded the cavity of a hollowed graphite electrode with red phosphorus (Figure S16) and performed an analogous oxidative LSV sweep as soon as it was lowered into the electrolyte, exposing the electrode to non-Faradaically generated P_4_ and allowing us to directly probe the required potential for back-oxidation in this system. With this red phosphorus-dosed electrode, we observe an open-circuit potential of 2.38 V vs Na/Na^+^ (Figure S17), and a positive-going scan from this potential reveals a large anodic wave we attribute to the reoxidation of P_4_ to phosphate (Figure 4, black trace). These data allow us to estimate a PO_3_^–^ ⇌ P_4_ equilibrium potential of approximately 2.4 V vs Na/Na^+^ in a metaphosphate melt, and highlight that P_4_ electrogeneration occurs near the thermodynamic limit under these conditions. This product-dosing methodology allows for steady-state voltages to be reported as true overpotentials η (Figure 5) and may serve as a general method for directly measuring reversible potentials in exotic reaction media in which equilibrium potentials cannot be readily calculated from thermochemical reference tables.

As previously mentioned, the reduction of PO_3_^–^ to P_4_ generates Lux basic oxide equivalents, which are consumed in this system via cleavage of Lux acidic phosphoryl anhydride linkages in the electrolyte (Figure S19). To assess the kinetic role of these linkages in facilitating PRR, we synthesized a series of melts of varying anhydride concentration, ranging from 5.4 to 9.8 mol kg^–1^, by dissolving sodium orthophosphate in pure sodium trimetaphosphate. These concentrations were chosen to correspond to the anhydride concentrations of pure sodium tripolyphosphate, tetrapolyphosphate, decapolyphosphate, and metaphosphate (Table S6). We then examined the electrochemical behavior of these melts by cyclic voltammetry and chronopotentiometry.

Assessing electrochemical potentials across electrolytes with varying Lux acidities requires a reference electrode that is independent of the Lux acidity of the medium. Given the demonstrated electroactivity of graphitic carbon electrodes in phosphate melts, it was plausible that any dependence of PRR on Lux acidity would induce a comparable drift in the carbon pseudoreference potential. Indeed, when referenced against a graphite electrode, we found the voltammetric features roughly overlaid across the entire Lux acidity range probed. As previously mentioned, we corrected for this oxide dependence of the carbon electrode by calibrating the pseudoreference potential against an oxide-independent Na/Na^+^ reference (Figure 5, top inset). Applying this calibration, CV data across these electrolytes are plotted on a common Na/Na^+^ reference scale (Figure 5, top). These data reveal a positive shift in the PRR wave by ∼250 mV as the anhydride concentration increases from 5.4 to 9.8 mol kg^–1^. In effect, as the Lux acidity of the medium increases with rising anhydride fraction, PRR becomes increasingly favorable—comparable to the potential shift observed for proton-coupled electrochemical reactions in water as the pH decreases.

This shift is also observed for the anodic oxidation of carbon, indicating that it, too, is sensitive to the Lux acidity of the melt. We stress that the observed dependence on Lux acidity is only apparent upon calibration to the oxide-independent Na/Na^+^ reference electrode. This is directly analogous to the requirement for a pH-independent reference electrode (e.g., Ag/AgCl) in order to identify pH-dependent electrochemical trends in aqueous media. These data highlight that phosphate electroreduction is highly sensitive to the Lux acidity of the melt, providing a valuable handle that can be tuned to optimize the efficiency and durability of P_4_ electrosynthesis. In accordance with Scheme 1, we find that addition of phosphoric acid to an anhydride-depleted melt restores the Lux acidity of the medium and regenerates the electrochemical features of a freshly prepared trimetaphosphate melt (see Section S2.1 and Figures S20 and S21). Varying the Lux acidity also serves to modulate the solubility of spectator ions such as Ca^2+^ (see Section S2.3). Modulating the Lux acidity of alternative fluxes such as CaCl_2_ could additionally serve to promote P_4_ electrosynthesis across a wide range of reaction media.^24,35^

Insights into the mechanism of PRR may be gleaned by analyzing the steady-state current-overpotential (Tafel) behavior of the system. In anhydride-rich pure trimetaphosphate melts (Figure 5, bottom, black trace), we observe a roughly log–linear scaling in current and overpotential across a range of 3.5 orders of magnitude in current density, with current densities in excess of −300 mA cm^–2^ accessible at modest overpotentials of −500 mV. In the pure metaphosphate melt we observe a Tafel slope of 150 mV dec^–1^, corresponding to an empirical transfer coefficient of *b* ≈ 1.4. This accords with an ideal transfer coefficient of 1.5, for a mechanism involving a reversible one-electron transfer followed by a rate-limiting one-electron transfer step.^36,37^ In the context of phosphate reduction, a mechanistic sequence involving quasiequilibrium P^V^ to P^IV^ reduction, followed by rate-limiting P^IV^ to P^III^ reduction, would be consistent with the observed Tafel behavior. This electrogenerated P^III^ species may then proceed to P^(0)^ via rapid electroreduction or disproportionation.^38^

The observed Tafel behavior also demonstrates the primacy of the Lux acidity in this system. Within error, the Tafel plots overlay at low overpotential, but a progressive deviation from linearity is observed at higher overpotentials and current densities as the Lux acidity of the melt decreases (black to red to blue to green in Figure 5, bottom). For the melts with the lowest anhydride population, the Tafel plots recoalesce at a −1.5 V overpotential. In line with literature reports and thermochemical reference data, we impute this high overpotential region to the reduction of orthophosphate generated at the electrode surface.^21,22,39−45^ Thus, we attribute this transition in the Tafel behavior to the local consumption of anhydrides near the electrode surface, a phenomenon that occurs at lower current densities for melts with lower bulk Lux acidities. We note that while a high anhydride population permits P_4_ production at reduced overpotentials, any anode reaction must regenerate these anhydride linkages, potentially introducing a countervailing overpotential penalty on the anode. However, just as the kinetics of aqueous half reactions such as hydrogen evolution display complex nonmonotonic trends with pH,^46^ we too find that graphite oxidation is less sensitive to changes in Lux acidity (see Section S2.5 and Figure S25) than PRR. Notwithstanding, for the PRR, the anhydride linkages in these melts play a key promoting role by functioning as preassociated Lewis acids, promoting P–O cleavage and dramatically reducing the overpotential for P_4_ synthesis by nearly a full volt, by direct analogy to prior reports of Lewis acid-promoted reduction of organophosphine oxides.^47^

## Conclusions

This work establishes a foundation for investigating phosphate electroreduction in molten salts. We present a set of electroanalytical methodologies capable of quantifying the overpotential, selectivity, and efficiency of phosphate reduction, and use these tools to compare reactivity across a continuum of electrolyte compositions with varying Lux acidity. These studies reveal that Lux acidic phosphoryl anhydride moieties are powerful activators of P–O bonds, enabling the reversible 20-electron reduction of phosphate to white phosphorus and the sustained production of P_4_ at high Faradaic efficiency and low overpotentials.

The versatility of the condensed phosphate electrolyte system opens the door to numerous future studies toward decarbonization of the phosphorus industry. The fundamental insights presented here about the role of Lux acidity in phosphate electroreduction suggest that phosphoryl anhydride moieties may serve to promote phosphate reduction across a wide array of electrolyte compositions.^21,22,24,35,48^ Complementarily, the full decarbonization of P_4_ electrosynthesis (like many industrial electrolyses, including the Hall process) requires the development of stable and efficient oxygen-evolving anodes. Analogous to the promotion of oxygen evolution in alkaline aqueous electrolytes,^49,50^ oxygen evolution in molten salts may be promoted by modulating the Lux basicity of the electrolyte,^23^ providing a powerful handle for balancing anode and cathode efficiency, selectivity, and durability. Cation studies present an orthogonal avenue of inquiry; the melt cation composition may be varied to tune half-reaction selectivity, while eutectic cation mixtures could serve to further reduce the operating temperature of phosphorus electrosynthesis and enable combined solar-thermal routes to low-carbon P_4_ synthesis.^19,51,52^ Given the imperative to decarbonize all sectors of the chemical industry, continued fundamental study of electrolytic phosphorus synthesis may one day enable replacement of the thermal process altogether.

## Supporting Information Available

Additional experimental details, materials, and methods, including photographs of experimental setup, reactor design, electrochemical and chemical analytical techniques, as well as supplemental experiments to demonstrate electrolyte replenishment, the impact of impurities, the solubility of calcium, and long-term electrolysis (PDF)

## Abbreviations

PRR: phosphate reduction reaction
W.E.: working electrode
R.E.: reference electrode
C.E.: counter electrode
GC: gas chromatography
CV: cyclic voltammetry
NMR: nuclear magnetic resonance
OCP: open-circuit potential
LSV: linear-sweep voltammetry
