# Supplementary material for: Electrolytic Synthesis of White Phosphorus Is Promoted in Oxide-Deficient Molten Salts
Source: ACS Cent Sci. 2023 Feb 21;9(3):373–80. doi: 10.1021/acscentsci.2c01336 (PMC10037495; doi:10.1021/acscentsci.2c01336)
Supplement: Supplementary file 1 — oc2c01336_si_001.pdf [file oc2c01336_si_001.pdf]

Supporting Information for:

Electrolytic Synthesis of White Phosphorus is Promoted in Oxide-Deficient Molten Salts

Jonathan F. Melville†, Andrew J. Licini†, Yogesh Surendranath*

Corresponding Author

***Yogesh Surendranath** – *Department of Chemistry, Massachusetts Institute of Technology, Cambridge, Massachusetts 02139, United States;* orcid.org/0000-0003-1016-3420; Email: yogeshs@mit.edu

Authors

†**Jonathan F. Melville** – *Department of Chemistry, Massachusetts Institute of Technology, Cambridge, Massachusetts 02139, United States*
†**Andrew J. Licini** – *Department of Chemistry, Massachusetts Institute of Technology, Cambridge, Massachusetts 02139, United States*

*Corresponding author.
†These authors contributed equally.

This PDF file includes:

Materials and Methods
Supplementary Experiments
Supplementary References
Figs. S1 to S23
Tables S1 to S8

Contents

**Section S1**	Materials and Methods	S4
**S1.1**	High-Temperature Electroanalytical Reactor Design	S4
	**S1.1a –** Crucible Selection	S8
	**S1.1b –** Counter Compartment	S8
	**S1.1c –** Working Electrode	S8
	**S1.1d –** Counter Electrode	S10
	**S1.1e –** Reference Electrodes	S10
	**S1.1f –** Graphite Pseudoreference	S12
	**S1.1g –** Liquid Sodium Electrode	S12
**S1.2**	Computational Modelling of Cyclic Voltammetry	S12
**S1.3**	Product Detection and Quantitation	S21
	**S1.3a –** Gas Flowthrough Setup	S21
	**S1.3b –** Product Capture	S22
**S1.4**	^{31}P NMR Analysis and Quantitation	S25
	**S1.4a –** ^{31}P NMR Identification of P_4	S25
	**S1.4b –** Quantitative ^{31}P NMR	S25
	**S1.4c –** Calculation of Faradaic Efficiency	S28
**S1.5**	Experimental Electrochemical Studies	S29
	**S1.5a –** Calculation of Electrochemical Surface Area	S29
	**S1.5b –** Collection of Cyclic Voltammetry Data	S32
	**S1.5c –** Current-Overpotential Studies	S32
	**S1.5d –** Collection of Galvanostatic Tafel Data	S32
	**S1.5e –** Determination of Overpotential	S32
	**S1.5f –** Methodology for Tafel Analysis	S36
**S1.6**	Gas Chromatography of Gas Outflow Streams	S36
**S1.7**	Composition and Structure of Molten Condensed Phosphates	S37
	**S1.7a –** Preparation of Selected Condensed Phosphate Mixtures	S40
**Section S2**	Supplemental Experiments	S42
**S2.1**	Replenishment of Anhydride-Depleted Melt with Phosphoric Acid	S42
	**S2.1a –** Electrochemical Characterization of Anhydride-Depleted Polyphosphate Melt	S42
	**S2.1b –** Phosphoric Acid Addition and Dehydration	S42
	**S2.1c –** Electrochemical Characterization Following Addition of Phosphoric Acid	S42
	**S2.1d –** ^{31}P NMR Characterization Prior to and Following Phosphoric Acid Addition	S44
**S2.2**	Electrolysis of “Impure” Phosphoric Acid	S46
**S2.3**	Solubility of Calcium in Condensed Phosphate Melts	S49
**S2.4**	Longer-Term Electrolysis	S52
**S2.5**	Steady-State Kinetics of Graphite Rod Oxidation	S54
**S2.6**	XPS of Graphite Cathode Tips	S56
**Section S3**	Supplemental References	S58

		Page
**Figure S1**	Cutaway render of assembled high-temperature electrochemical reactor	S5
**Figure S2**	Exploded render of high-temperature reactor	S6
**Figure S3**	Diagram of molten-salt electrolysis setup	S7
**Figure S4**	Photos of graphite working-electrode morphologies	S9
**Figure S5**	Open-circuit potential trace of graphite pseudoreference electrode	S11
**Figure S6**	Photo of Na/Na+ reference, with blowup of tip structure	S12
**Figure S7**	Simulated CVs for a putative EEC phosphate reduction mechanism	S16
**Figure S8**	Simulated CVs for a putative EECC phosphate reduction mechanism	S20
**Figure S9**	Photos of electrogenerated white and red phosphorus	S23
**Figure S10**	Photos of red phosphorus accretion on interior reactor surfaces	S24
**Figure S11**	^{31}P NMR spectrum of electrogenerated P_4 in CS_2	S26
**Figure S12**	^{31}P NMR spectrum of dissolved polyphosphates from bleach trap	S27
**Figure S13**	Representative capacitive CVs for ECSA determination of graphite	S30
**Figure S14**	Representative linear fit for ECSA determination of graphite	S31
**Figure S15**	LSV traces of hollowed graphite electrode containing electrogenerated P_4	S33
**Figure S16**	Photo of hollowed graphite electrode dosed with red phosphorus	S34
**Figure S17**	OCP traces of a various graphite electrode morphologies	S35
**Figure S18**	Classifications of condensed phosphate species	S38
**Figure S19**	Representative mechanism for oxide cleavage of phosphoryl anhydrides	S39
**Figure S20**	CVs before and after restoration of an anhydride-depleted melt by H_3PO_4	S43
**Figure S21**	^{31}P NMR spectrum of samples before and after H_3PO_4 restoration	S45
**Figure S22**	CV of a $Na_3(PO_3)_3$ melt modelling impurities from wet-process H_3PO_4	S48
**Figure S23**	Photographs of molten swabs from $Na_3(PO_3)_3$-$Ca_3(PO_4)_2$ mixtures	S51
**Figure S24**	CP of a long-term electrolysis using graphite rods in $Na_3(PO_3)_3$	S53
**Figure S25**	Steady-state CP data for graphite rod oxidation in $Na_3(PO_3)_3$, $Na_5P_3O_{10}$	S55
**Figure S26**	Steady-state CP data for graphite rod oxidation in $Na_3(PO_3)_3$, $Na_5P_3O_{10}$	S57
**Table S1**	Input parameters for simulated CVs by putative EEC mechanism	S14
**Table S2**	Simulation parameters for simulated CVs by putative EEC mechanism	S15
**Table S3**	Input parameters for simulated CVs by putative EECC mechanism	S18
**Table S4**	Simulation parameters for simulated CVs by putative EECC mechanism	S19
**Table S5**	Phosphate species and formulae by oxide and anhydride content	S39
**Table S6**	Selected condensed phosphate species investigated in this study	S41
**Table S7**	Masses for a $Na_3(PO_3)_3$ melt modelling impurities from wet H_3PO_4	S47
**Table S8**	Compositions of $Na_3(PO_3)_3$-$Ca_3(PO_4)_2$ mixtures and solubility findings	S50

Section S1 – Materials and Methods

S1.1 – High-Temperature Electroanalytical Reactor Design

An electrochemical reactor (**Figure 2**, **Figures S1-S3**) was designed from the ground up to accommodate the electroanalysis of a molten-salt system while maintaining separation of cathodic and anodic gas flow streams. The main reactor body consisted of a closed-end alumina tube (designated "Alumina tube, one-end-closed, OD60.33mm*ID52mm*L279.4mm (OD+/-4%, ID+3/-0)" by AdValue Technologies), to which a custom-machined stainless-steel cap with four Swagelok™ Ultra-Torr® fittings could be attached. These fittings, as well as the junction between the reactor head and body, were sealed with DuPont™ Kalrez® perfluoroelastomer O-rings (temperature rated to 325 °C). Of the four fittings, three (of 1/4" diameter) held alumina-sheathed electrodes, while the fourth (of 3/4" diameter) held the isolated counter compartment and electrode. Careful management of the temperature of these O-rings, accomplished by glass insulation wool and Variac-controlled heating tape, is essential to reconcile the thermal rating of these O-rings with the temperatures of reaction; at low temperatures, P_4 will collect on the reactor head and interior instead of venting through the cold trap, while at high temperatures the O-rings will melt, eventually compromising the reactor seal. The furnace itself was designed to fit a CF56622C Lindberg/Blue M™ top-loading Crucible Furnace (now discontinued) as controlled by a CC58114C-1 Lindberg/Blue M™ Furnace Controller.

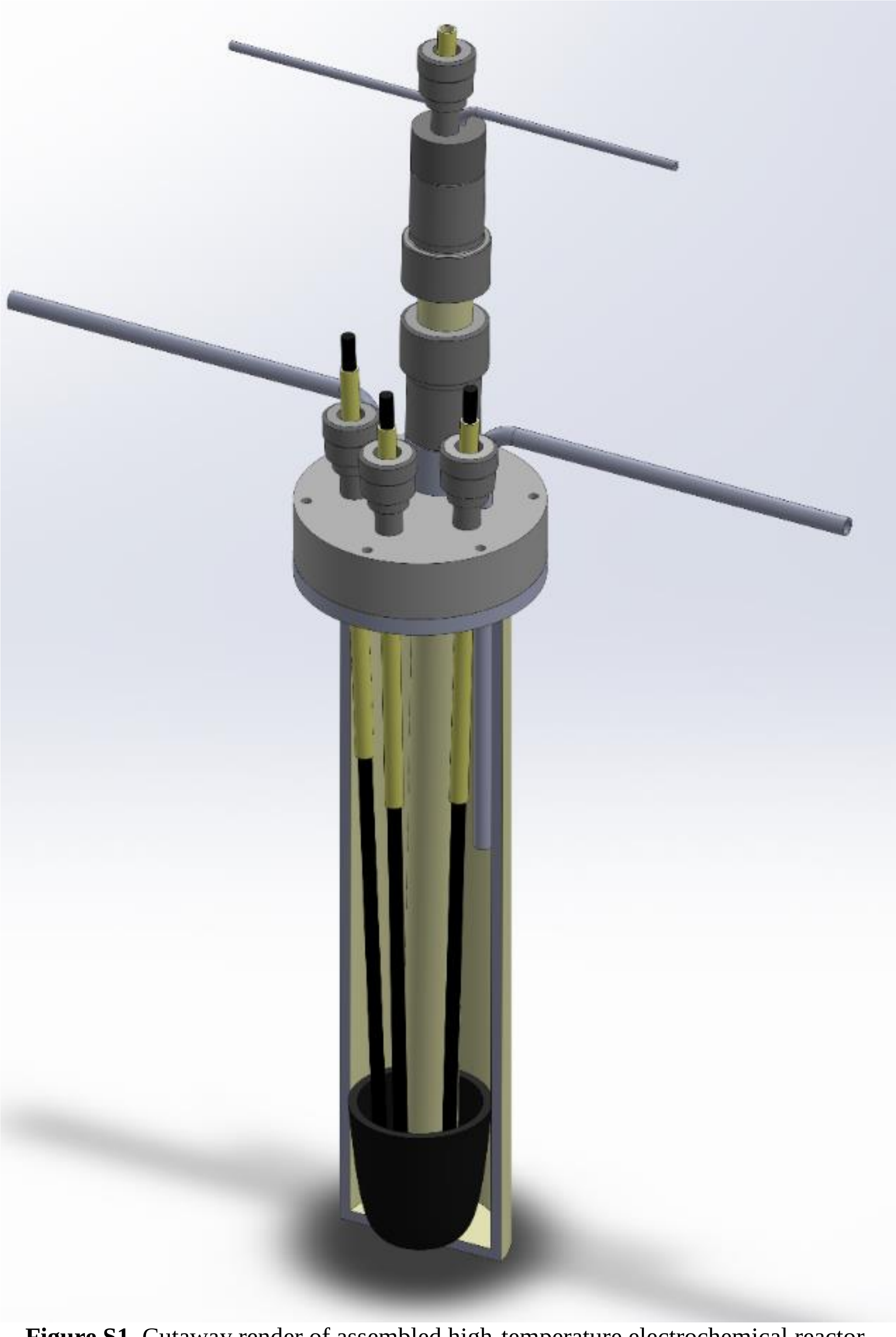

Figure S1. Cutaway render of assembled high-temperature electrochemical reactor.

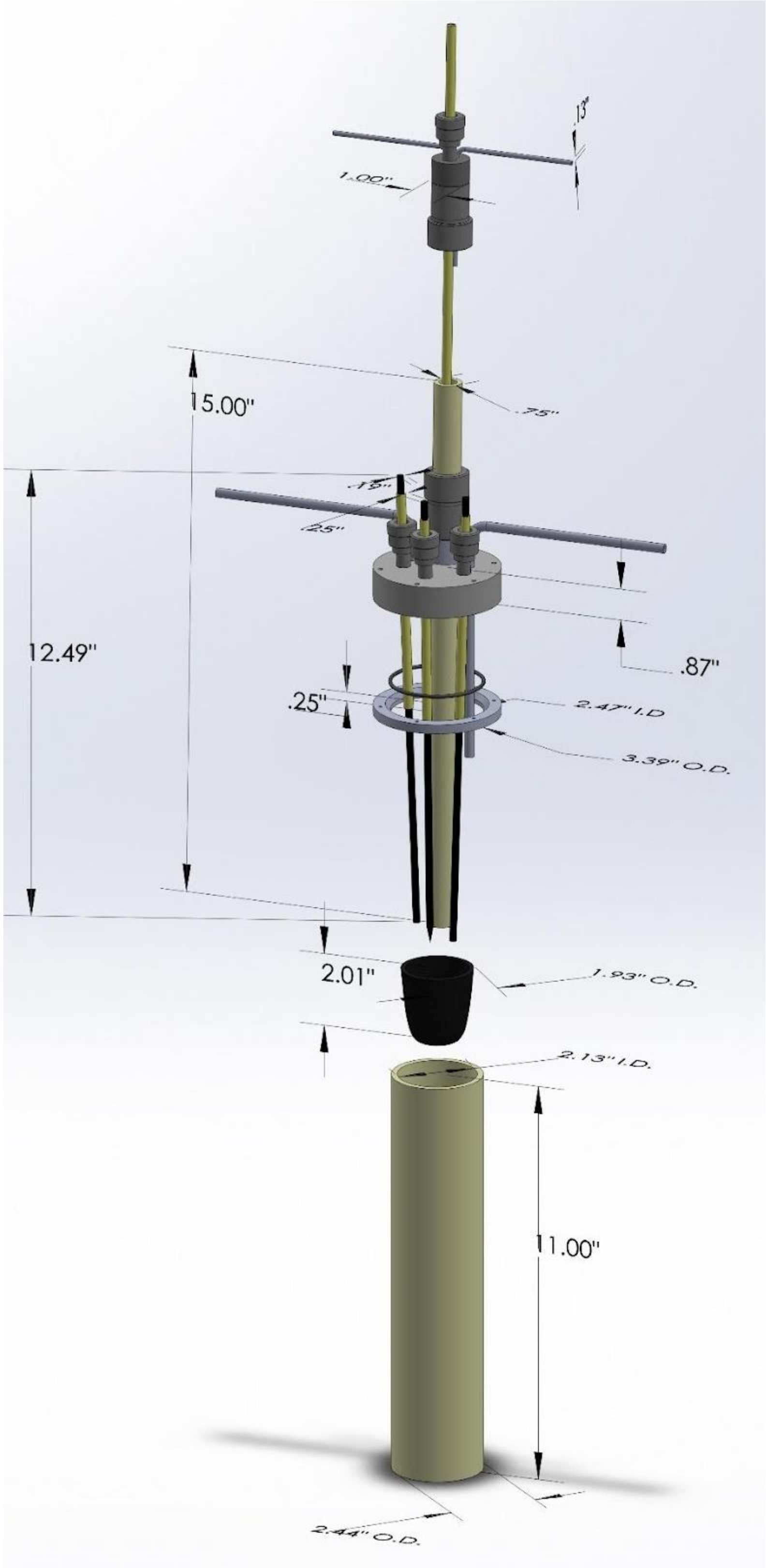

Figure S2. Exploded render of high-temperature reactor, including component dimensions.

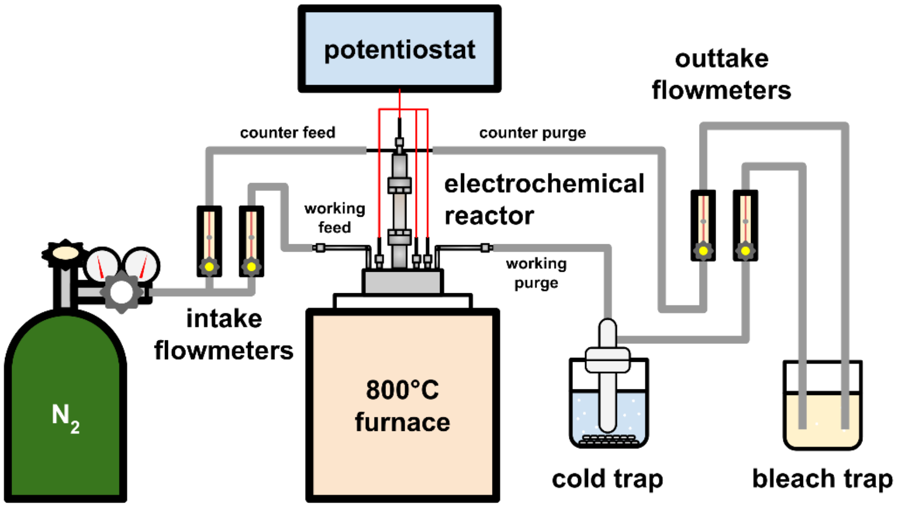

Figure S3. Diagram of molten-salt electrolysis setup configured for P_4 capture and quantitation, including separated cathodic and anodic N_2 gas flow streams.

S1.1a – Crucible Selection

The bottom of the electrochemical reactor contained a conical glassy carbon crucible (60 mL, 52 mm outer diameter (OD), part #39006KT from Alfa Aesar), so chosen for its inertness to the metaphosphate melt and the temperatures of reaction while under inert atmosphere. Between experiments, the crucible was polished with 800 grit sandpaper and an alumina slurry (1 micron deagglomerated hexagonal alumina powder, part #PSI-5601-5 by Precision Surfaces International, Inc.), then rinsed with reagent grade water (Millipore Type 1, 18.2 MΩ·cm resistivity). This polishing procedure was necessary to ensure that the fused melts could be easily separated from the crucible upon cooling following a reaction run.

S1.1b – Counter Compartment

For all experiments conducted in this study, the counter compartment consisted of a single-bore alumina tube (3/4" OD, 1/2" inner diameter (ID), 18" overall length, part #AL-T-N3/4-N1/2-18 by AdValue Technologies). Following the conclusion of these experiments, it was discovered that a clear fused quartz tube (19 mm OD, 15 mm ID, 1219 mm length, part #FQ-T-19-15-4 by AdValue Technologies, subsequently cut into 16"-long segments) was better suited to isolate the counter compartment. Quartz displayed enhanced resistance to corrosion in the presence of the molten metaphosphate electrolyte and its transparency allowed for visual inspection of the electrical contact made to the counter electrode.

S1.1c – Working Electrode

Unless indicated otherwise, the working electrode for all experiments was a graphite rod (Spectro-Grade: total impurity level < 2 ppm, individual element impurity levels < 1 ppm, 3/16 ” diameter, 12” length, part # 70231 by Electron Microscopy Sciences) with the end sharpened to a point in a pencil sharpener (**Figure S4**, center) and subsequently polished clean by rubbing with a Kimwipe™ to exfoliate potential trace metal impurities. In experiments which utilized a hollowed-electrode morphology, the end of a graphite rod (Spectro-Grade, 3/16 ” diameter, 12” length, part # 70231 by Electron Microscopy Sciences) was carved into a conical hollow using the corner of a razor blade (**Figure S4**, right), and subsequently polished clean by rubbing with a Kimwipe to exfoliate trace metal impurities. To prevent shorting of the electrode against the conductive reactor head, a single-bore alumina tube (1/4” OD, 3/16” ID, part #AL-T-N1/4-N3/16-12 by AdValue Technologies, cut to a length of approximately 4 inches) was used as an electrode sheath. Graphite electrodes were flame-sealed to the electrode sheath by using a propane blowtorch to melt a pea-sized volume of sodium metaphosphate salt, which was then dabbed onto the junction of the alumina sheath and the graphite electrode, adhering the two together.

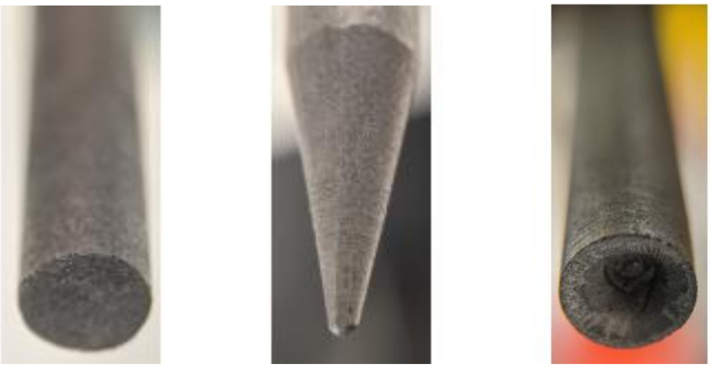

Figure S4. Graphite working-electrode morphologies employed in the study: (a) an unaltered rod (b) a sharpened rod to facilitate release of electrogenerated gas bubbles, and (c) a rod modified with a concave depression to trap P_4 bubbles.

S1.1d – Counter Electrode

Unless indicated otherwise, the counter electrode for all experiments was a graphite rod (Spectro-Grade, 1/4” diameter, 12” length, part #70230 by Electron Microscopy Sciences™), the end polished by rubbing against a Kimwipe™ to exfoliate potential impurities. As this work focuses primarily on investigating the cathodic reduction of phosphate to elemental phosphorus, graphite was chosen as a sacrificial anode for these studies. The development of corrosion-resistant oxygen evolving anodes for high-temperature molten salt electrolysis is the subject of active ongoing investigations (*53–57*).

S1.1e – Reference Electrodes

All electrolytic experiments were performed using a graphite pseudoreference electrode. We found that this pseudoreference maintains a stable potential in a quiescent melt over long timescales, but the precise value of its potential was found to vary both within an experiment (due to local variations in melt composition induced by electrochemical action) and between experiments (due to alterations in the bulk composition of the melt). Thus, the graphite pseudoreference electrode was calibrated before and after each experiment against a liquid-sodium Na/Na^+ absolute reference electrode (S4 Na/Al by Ionotec Ltd.). The solid-electrolyte junction of the sodium reference electrode was subject to slow corrosion in the metaphosphate electrolyte precluding its direct use as a full-time reference electrode. This calibration procedure minimized exposure of the Na/Na+ reference to the corrosive medium, but still allowed for comparison of the potentials across a variety of melt compositions. Further experimental details about the graphite pseudoreference and the Na/Na^+ reference are provided below (*31*).

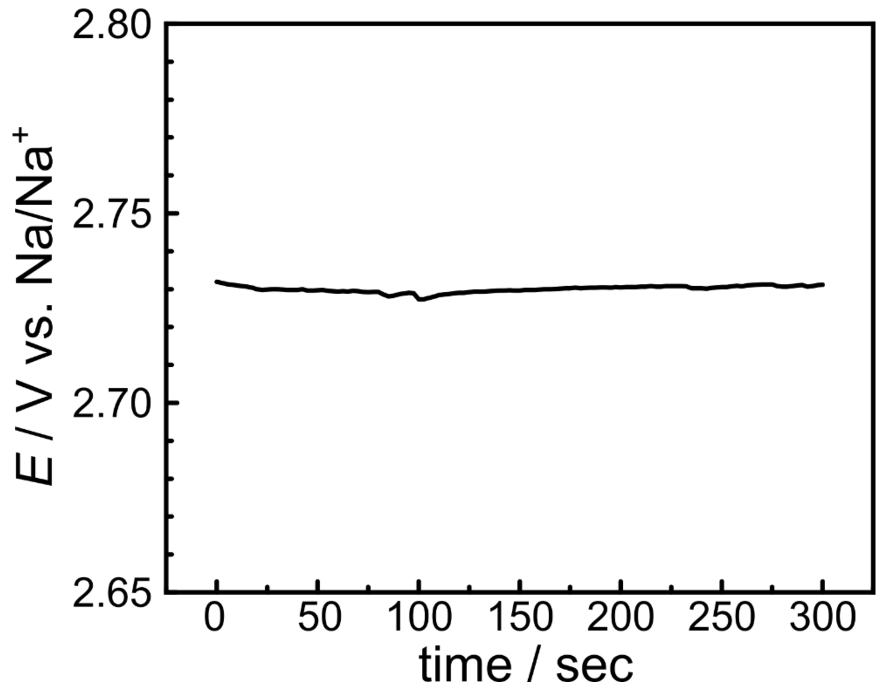

Figure S5. 5-minute open-circuit potential trace of graphite pseudoreference electrode against a Na/Na^+ reference electrode.

S1.1f – Graphite Pseudoreference

Unless indicated otherwise, the reference electrode for all experiments was a graphite rod (Spectro-Grade, 3/16” diameter, 12” length, part #70231 by Electron Microscopy Sciences™), the end polished clean by a Kimwipe™ to exfoliate potential impurities. To prevent shorting of the electrode against the conductive reactor head, a single-bore alumina tube (1/4” OD, 3/16” ID, part #AL-T-N1/4-N3/16-12 by AdValue Technologies, cut to a length of approximately 4 inches) was used as an electrode sheath. The graphite electrode was flame-sealed to the electrode sheath by using a propane blowtorch to melt a pea-sized volume of sodium metaphosphate salt, which was then dabbed onto the junction of the alumina sheath and the graphite electrode, adhering the two together.

S1.1g – Liquid Sodium Electrode

The liquid sodium reference electrode was custom-ordered from Ionotec Ltd. under the product designation “S4 Na/Al: Na beta-alumina reference electrode, 4.0 mm ID x 70 mm length x 0.6 mm wall thickness” and used as received. The potential pinned by the reference is the equilibrium between the sodium metal and the sodium ions within the membrane, as depicted by **Figure S6**.

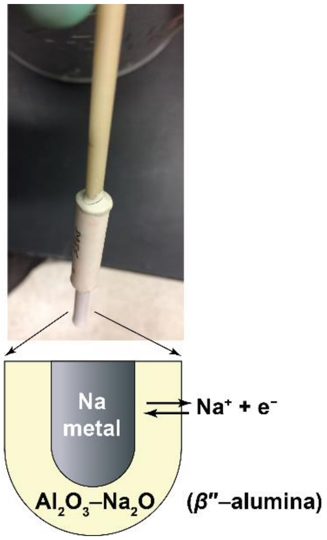

Figure S6. Photograph of Na/Na^+ reference electrode, with blowup indicating the structure of the electrochemically active sodium metal tip encased in a sodium ion-selective β″-alumina ceramic membrane.

S1.2 – Computational Modeling of Cyclic Voltammetry

To assess the plausibility of our putative mechanism and reconcile the activation-controlled current-overpotential relation in **Figure 3** with the diffusion-controlled voltammograms seen in **Figure 5**, we employed Gamry™ Elchsoft™ DigiElch® Electrochemical Simulation Software to simulate hypothetical cyclic voltammetric behavior, as seen in **Figure S7** and **Figure S8**. In an initial simulation, we modeled an EEC mechanism consisting of two consecutive fast electron-transfer steps followed by a comparatively slow chemical disproportionation. The first electron transfer (representing the reduction of $P^{V} \rightarrow P^{IV}$) occurred at an arbitrarily-selected potential of $E°$ = 0 V and a rapid $k_s = 1 \times 10^5$ cm·s^{-1}, while the second electron transfer (representing the reduction

of $P^{IV} \rightarrow P^{III}$) was chosen to occur at $E°$ = −0.1 V with a slightly slower $k_s = 1 \times 10^4$ cm·s^{-1}; both reactions utilized a default symmetry factor of β = 0.5. The subsequent chemical reaction (representing the disproportionation of $P^{III} \rightarrow P^{(0)}$) was modeled with $k_f = 1\times10^{-4}$ and K_{eq} = 1. To represent the metaphosphate melt, the starting conditions for the simulation began at a concentration of 24 M for the simulated P^V species, which possessed a rapid diffusion coefficient of 10 cm^2·s^{-1}. All other species began at a starting concentration of zero; the P^{IV} and P^{III} species were assigned a sluggish diffusion coefficient of 1×10^{-20} cm^2·s^{-1} to represent the likely surface-bound nature of these partially reduced intermediates, while the terminal $P^{(0)}$ species had a modestly faster diffusion coefficient of 1×10^{-5} cm^2·s^{-1} to represent P_4 bubbling in the viscous electrolyte media.

Table S1. Input parameters for simulation of phosphate reduction to elemental phosphorus via a simulated EEC mechanism (**Figure S7**).

Reaction	Model	E^0 (V) / K_{eq}	ET Transfer Coefficient (α)	k_s (cm/s) / k_f
$P^V + e^- \rightleftharpoons P^{IV}$	Butler-Volmer	0	0.5	1×10^5
$P^{IV} + e^- \rightleftharpoons P^{III}$	Butler-Volmer	−0.1	0.5	1×10^4
$P^{III} \rightleftharpoons P^{(0)}$	Chemical Equilibrium	1	N/A	1×10^{-5}

Table S2. Simulation parameters for simulation of phosphate reduction to elemental phosphorus via a simulated EEC mechanism (**Figure S7**).

Experimental Parameters:

E_{start}	0.1 V
E_{switch}	−0.2 V
E_{end}	0.1 V
Scanrate(s)	100, 50, 25, 10 $mV \cdot s^{-1}$
Temperature	500 K
R_u	1 Ω
C_{dl}	1×10^{-5} F
Cycles	1

Electrode geometry	Planar
Area	1 cm^2
Diffusion	Semi-infinite 1D
Pre-equilibrium	All reactions

Species parameters:

$[P^{V}]$	24 M	1×10^{-5} $cm^2 \cdot s^{-1}$
$[P^{IV}]$	0 M	1×10^{-20} $cm^2 \cdot s^{-1}$
$[P^{III}]$	0 M	1×10^{-20} $cm^2 \cdot s^{-1}$
$[P^{(0)}]$	0 M	10 $cm^2 \cdot s^{-1}$

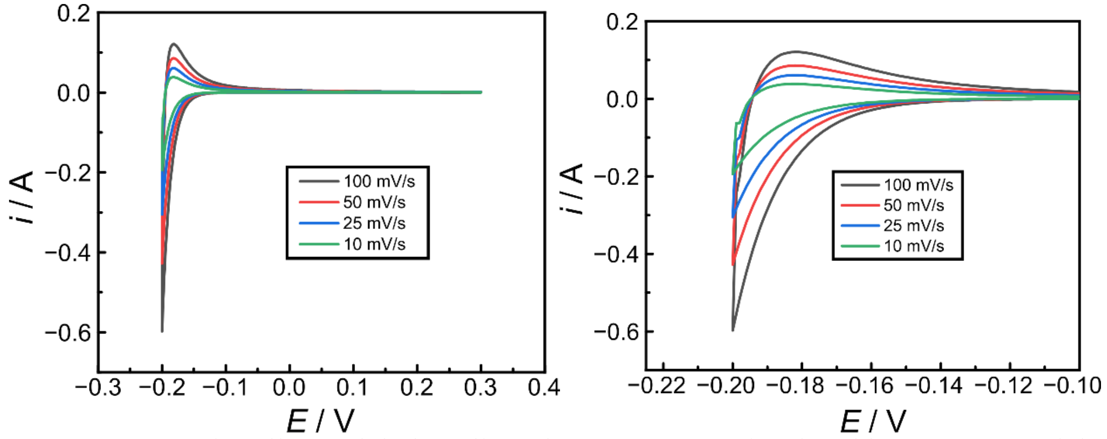

Figure S7. Computationally modeled cyclic voltammograms, simulated by Gamry™ Elchsoft™ DigiElch® Electrochemical Simulation Software, for a putative EEC phosphate reduction mechanism consisting of two consecutive rapid electron-transfer steps followed by a comparatively slow chemical disproportionation. (a) displays the full CV simulation, while (b) displays a zoomed view of the simulated reductive feature.

We also performed additional simulations (**Figure S8**) on a putative EECC mechanism consisting of two consecutive electron-transfer steps $P^{V} \rightarrow P^{IV} \rightarrow P^{III}$ followed by a chemical disproportionation to a surface-bound phosphorus species (denoted "red P") that proceeds towards a terminal white phosphorus product by a second chemical reaction. In this model, the partially reduced intermediates P^{IV} and P^{III} are *not* surface-bound; rather, the square-root scan-rate dependence is evinced by the surface-bound nature of the intermediary *chemically* generated red P intermediate.

Table S3. Input parameters for simulation of phosphate reduction to elemental phosphorus via a simulated EECC mechanism (**Figure S8**).

**Reaction**	**Model**	E^0 **(V) /** K_{eq}	**ET Transfer Coefficient (α)**	k_s **(cm/s) /** k_f
$P^V + e^- \rightleftharpoons P^{IV}$	Butler-Volmer	0	0.5	1×10^5
$P^{IV} + e^- \rightleftharpoons P^{III}$	Butler-Volmer	−0.05	0.5	1×10^4
$P^{III} \rightleftharpoons$ red P	Chemical Equilibrium	1	N/A	1
red P $\rightleftharpoons$ white P	Chemical Equilibrium	1	N/A	1

Table S4. Simulation parameters for simulation of phosphate reduction to elemental phosphorus via a simulated EECC mechanism (**Figure S8**).

Experimental Parameters:

E_{start}	0.1 V
E_{switch}	−0.1 V
E_{end}	0.1 V
Scanrate(s)	100, 50, 25, 10 $mV \cdot s^{-1}$
Temperature	500 K
R_u	1 Ω
C_{dl}	1×10^{-5} F
Cycles	1

Electrode geometry	Planar
Area	1 cm^2
Diffusion	Semi-infinite 1D
Pre-equilibrium	All reactions

Species parameters:

$[P^{V}]$	24 M	1×10^{-5} $cm^2 \cdot s^{-1}$
$[P^{IV}]$	0 M	1×10^{-5} $cm^2 \cdot s^{-1}$
$[P^{III}]$	0 M	1×10^{-5} $cm^2 \cdot s^{-1}$
red P	0 M	1×10^{-20} $cm^2 \cdot s^{-1}$
white P	0 M	10 $cm^2 \cdot s^{-1}$

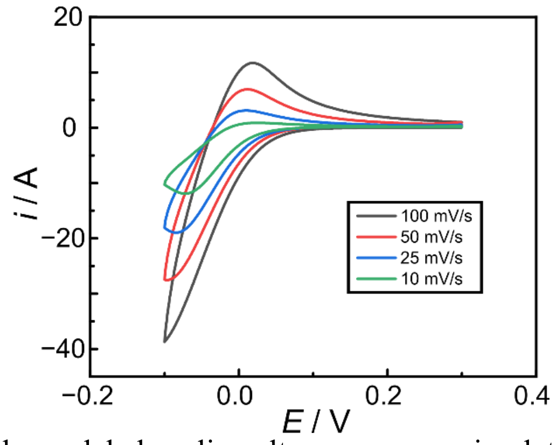

Figure S8. Computationally modeled cyclic voltammograms, simulated by Gamry™ Elchsoft™ DigiElch® Electrochemical Simulation Software, for a putative EECC phosphate reduction mechanism consisting of two consecutive electron-transfer steps, followed by a chemical disproportionation to a surface-bound phosphorus species that proceeds towards a terminal phosphorus product by a second chemical reaction.

The simulated voltammograms seen in **Figure S7** and **Figure S8** reproduce the profile and scan-rate dependence of the diffusion-controlled voltammograms seen in **Figure 3**. In particular, the simulation captures the squareroot scan-rate dependence of the reductive current densities as well as the presence of common potential corresponding to j=0 across all traces. **We stress that is qualitative similarity between simulated and experimental voltammograms should *not be taken* as an indication of the validity of the simulation parameters**; rather, these computations provide an explanation for the apparent contradiction between our observation of diffusion-controlled cyclic voltammograms (**Figure 3**) and the activation-controlled Tafel curves (**Figure 5**). In particular, the simulations indicate that an observed square-root scan-rate dependence in the CV may result not from diffusion limitation of a reactant species, but rather the build-up of reduced intermediates such as $P^{(III)}$ and $P^{(IV)}$ species in the reaction diffusion layer, a phenomenon which evinces itself if *any* electrochemically or chemically generated species following the initial phosphate reduction is bound to the electrode surface. This build-up may lead to a diffusion-limited back-oxidation current, which upon summing with a scan-rate independent reduction current, manifests as a diffusion-limited voltammogram. In particular, since $j_{\mathrm{tot}} = j_{\mathrm{red}} - j_{\mathrm{ox}}$, the lower j_{tot} observed at lower scan-rates is not due to reduced diffusion-limited j_{red}, but *increased* diffusion-limited j_{ox}. This phenomenon may be enabled by low gas solubility and product diffusion coefficients in the metaphosphate electrolyte, which means that slower scan rates have a greater buildup of reduced products near the electrode surface.

S1.3 – Product Detection and Quantitation

S1.3a – Gas Flowthrough Setup

In order to account for potential gas flow losses due to loss of O-ring seal integrity at temperature, a gas flowthrough setup (**Figure S3**) was constructed to enable quantitation of N_2 gas flow into and out of the electrochemical reactor, utilizing acrylic valved bead flowmeters (part #PB-3246040 by Cole-Parmer Scientific™) leading into and out of both the working and counter electrode compartments. Working compartment flowmeters were rated for between 40 to 500 standard cubic centimetres per minute (sccm), while counter compartment flowmeters were rated for between 10 and 50 sccm. At typical flowrates of 40 sccm through the counter compartment and 400 sccm through the working compartment, temperature effects due to volumetric expansion were found to be within the error of the system. In quantitating P_4 yield for calculating Faradaic efficiency (FE%), observed yield was normalized by working compartment gas outflow as a fraction of working compartment gas inflow. In order to verify that all nitrogen flow loss occurred after the nitrogen gas entered the cell headspace chambers rather than in the feed line junctions, an experiment was conducted where the cell was bypassed and the feed lines were connected directly to the exhaust lines via Swaglok seals. No nitrogen leaks were observed as judged by identical upstream and downstream volumetric flow rates and manual inspection via application of soapy solution to seal junctions. For analysis of counter electrode outflow streams, gas chromatography (GC) analysis was performed by injection into a Multi-Gas Analyzer (#3; SRI Instruments) equipped with a thermal conductivity detector, methanizer, and flame ionization detector in series after Molsieve 13x and Hayesep D Columns.

We note that gas losses between the inlet and outlet of the cell result principally from advective gas flow small but macroscopic gaps between the rough alumina cell components and the Kalrez rubber-based O-rings used in the Ultra-Torr fittings. The leaks are especially pronounced through

the 2.37-inch-diameter O-ring that seals the outer wall of the cell. We also note that Kalrez O-rings also have limited stability at the very high temperatures of our cell, contributing to these macroscopic leaks. The advection is induced by the positive pressure differential at which the cell is maintained relative to the outside environment to minimize the ingress of air into the electrochemical cell. Maintaining a positive pressure differential is key to the safe operation of the cell due to the pyrophoric nature of P_4. Although N_2 and P_4 have distinct diffusivities, advective transport is not dependent on the molecular mass of the gas and so, we expect P_4 losses to be in direct proportion to the overall gas losses in the cell.

S1.3b – Product Capture

Evolved phosphorus was collected from three primary locations: the cold trap, the bleach trap, and the reactor head. The cold trap, immersed in a dry ice/acetone bath at -78 °C, was the primary method of capture and the only method by which molecular P_4 could be collected. To mitigate photoconversion of P_4 to polymeric red phosphorus (**Figure S9**), the cold trap was wrapped in aluminum foil; following an experiment, the valves were sealed and the cold trap was pumped into a N_2 glovebox, within which P_4 samples for ^{31}P NMR could be prepared. The terminal bleach trap sometimes accumulated measurable quantities of phosphorus in the form of oxidized polyphosphate species, presumably formed by the oxidation of residual P_4 that exited the cold trap. Finally, polymeric red phosphorus deposits on the reactor interior (**Figure S10**), formed when the reactor head cooled below the 280.5 °C boiling point of P_4, were collected for quantification by oxidation and dissolution with bleach. As a control experiment, the reactor was brought to temperature and its graphite electrodes were submerged and held at open circuit potential for 12 hours. Under these conditions, no gaseous products were observed, as supported by the lack of condensate on the reactor head and the absence of observable products in the bleach or cold traps. This finding establishes that faradaic current is required for the generation of white phosphorus, and does not occur carbothermally under our conditions.

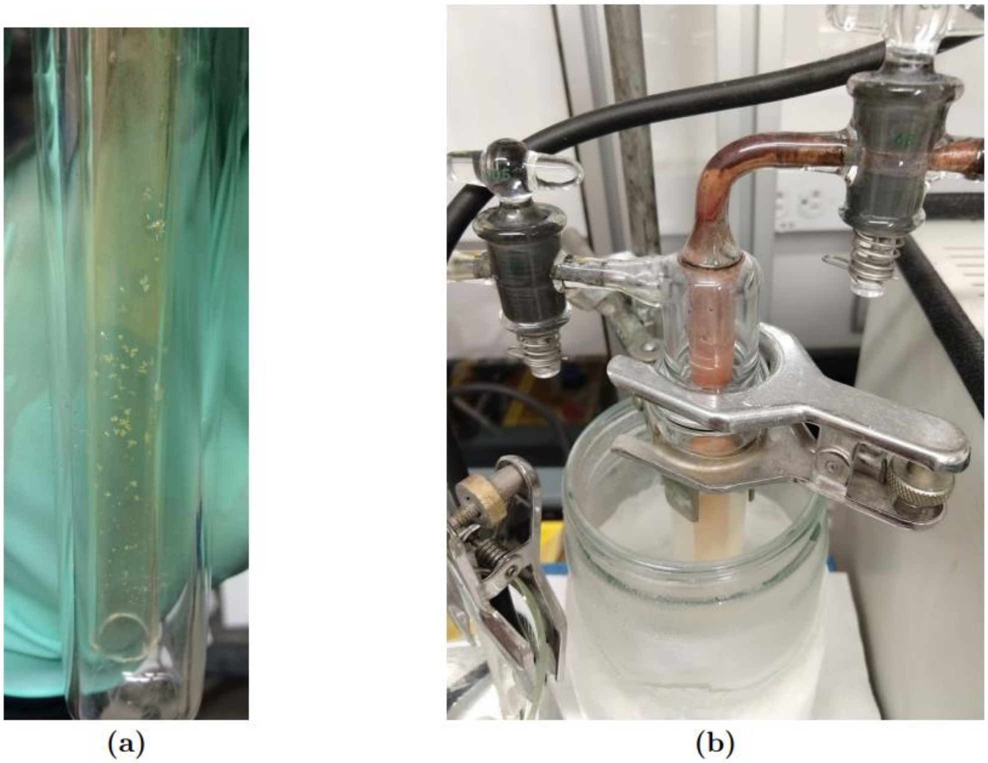

Figure S9. (a) Yellow-white crystals of white phosphorus are observed to form in the cold trap upon sustained electrolysis. (b) Upon exposure to light, electrogenerated white phosphorus will photoconvert to polymeric red phosphorus.

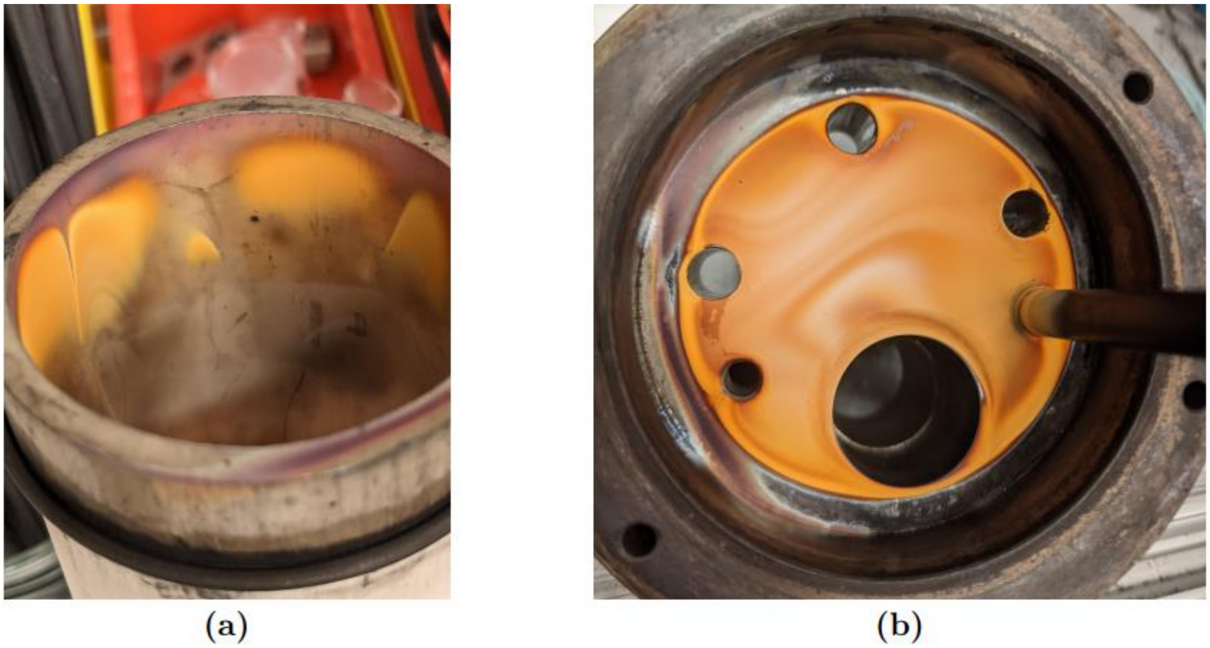

Figure S10. Post-electrolysis accumulation of red phosphorus on reactor body (a) and head (b) interiors.

S1.4 – ^{31}P NMR Analysis and Quantitation

^{31}P NMR spectra were recorded with either Bruker AVANCE-400 or Bruker AVANCENeo-500 spectrometers and processed using MestReNova software. In order to allow for accurate integration of phosphate peaks for quantification, all ^{31}P NMR spectra were recorded without proton or deuterium decoupling pulses. ^{31}P shifts are given in ppm with respect to externally referenced triphenylphosphine (TPP) ($\delta = -6.0$ ppm) or phosphonoacetic acid ($\delta = -17.0$ ppm). Coupling constants are reported as J-values in Hz.

S1.4a – ^{31}P NMR Identification of P_4

For the collection of ^{31}P NMR spectra of molecular P_4, such as that seen in **Figure 3**, the reactor cold trap (wrapped in aluminum foil) was sealed following a bulk electrolysis (typically around 50 $mA \cdot cm^{-2}$ for several hours). The cold trap was then pumped into a N_2 glovebox, where the contents of the trap were dissolved with approximately 2 mL carbon disulfide (CS_2) along with a known quantity of triphenylphosphine (as an internal standard) as well as approximately 25 mg (0.072 mmol) of chromium(III) acetylacetonate as a paramagnetic relaxation agent. The sample was then transferred to a J. Young tube for ^{31}P NMR analysis. To ensure full spin relaxation for quantitation, a delay time of 10.0 s was used.

S1.4b – Quantitative ^{31}P NMR

Quantitative ^{31}P NMR techniques were applied for the calculation of total reduced phosphorus yield. Phosphorus residues were collected from four primary locations: the cold trap, the bleach trap, surfaces within the reactor head, and the internal surfaces of the transfer lines. As we had already established the formation of P_4 with the preceding NMR in CS_2, for these experiments, we dissolve all products in a 7.4 % w/w aqueous solution of sodium hypochlorite to facilitate the quantitation of all phosphorus emanating from the reactor. This bleach solution was used to oxidize elemental red phosphorus deposits (**Figure S10**) to soluble ortho- and polyphosphate species. These products were then quantified by aqueous ^{31}P NMR against an internal standard of phosphonoacetic acid, again using a delay time of 10.0 s to ensure full spin relaxation for quantitation. For quantitation of phosphorus in the bleach trap, an aliquot was taken and analyzed against a phosphonoacetic acid internal standard to determine the concentration of phosphorus in the bleach trap, which was then multiplied by the volume of liquid in the bleach trap. The total phosphorus content in the bleach trap, cold trap, reactor head, and transfer lines were summed to determine the overall phosphorus yield, N_P.

A similar analysis process was applied to the cooled melt compacts, where the melt compacts at the bottom of the crucible were powdered and dissolved in D_2O milli-Q water. The subsequent solutions were then analyzed via aqueous ^{31}P NMR. Following both Tafel and bulk electrolysis trials, ^{31}P NMR of the electrolyte displays peaks in the −20 to 0 ppm range corresponding to P(V) species—specifically polyphosphate ‘middle’ groups, ‘end’ groups and orthophosphate. However, we do not observe any peaks at more positive chemical shifts in the P(III) region of the spectrum, suggesting the absence of any detectable accumulation of partially reduced phosphorus species.

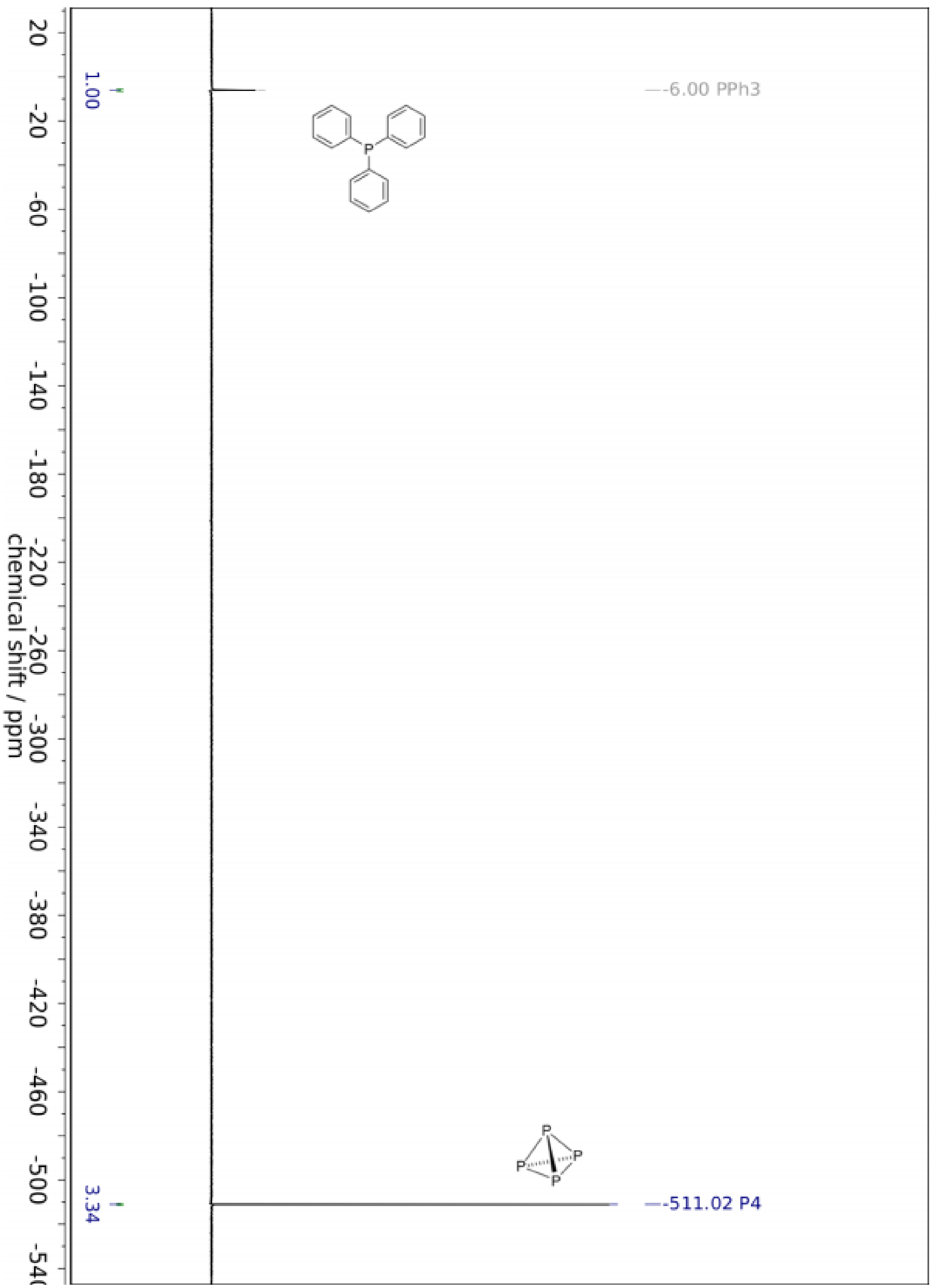

Figure S11. ^{31}P NMR spectrum of electrogenerated P_4 collected from cold trap, dissolved in CS_2 against a triphenylphosphine internal standard.

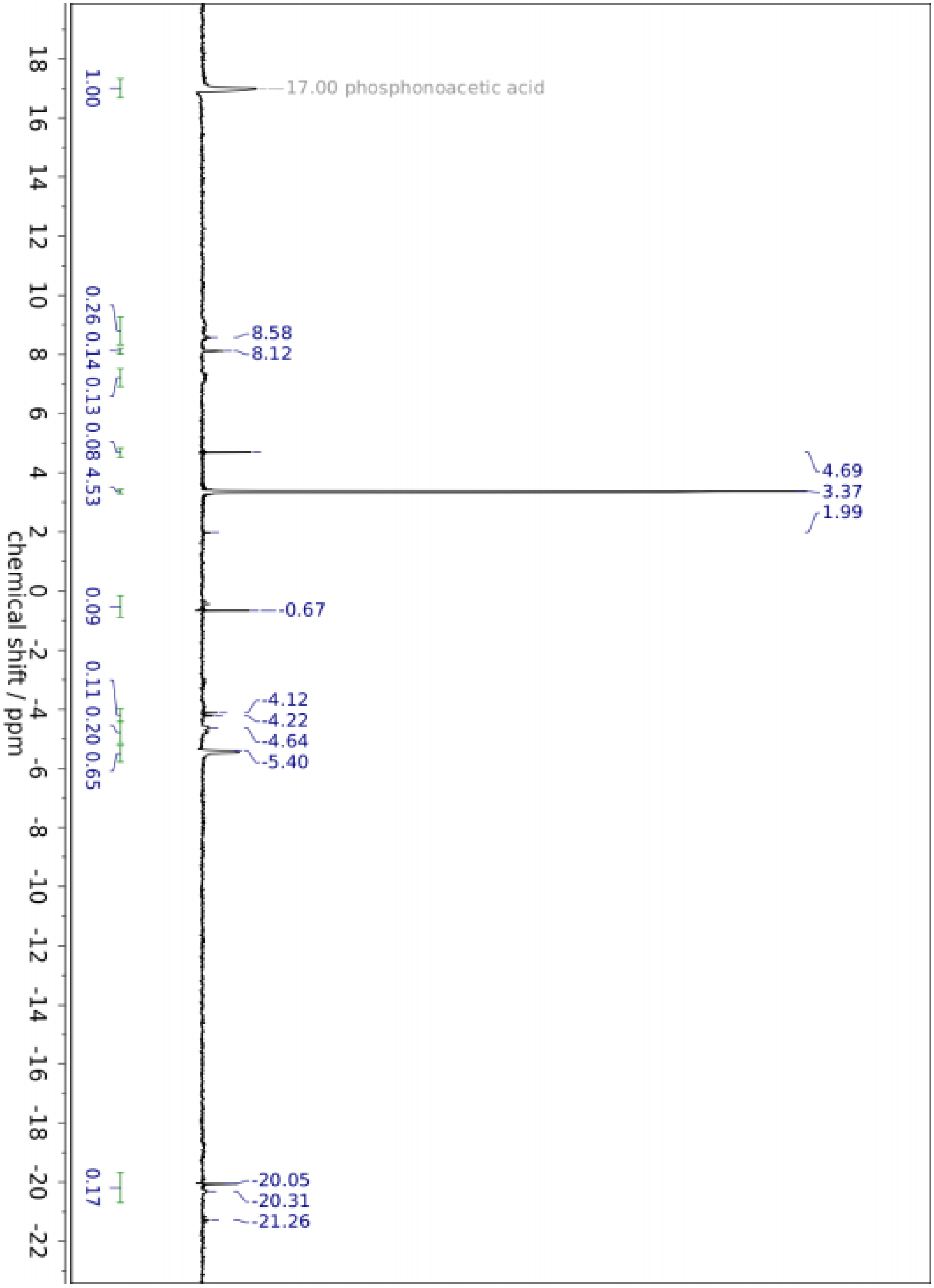

Figure S12. Representative quantitative ^{31}P NMR spectrum of bleach trap post-electrolysis. Integrals are normalized to phosphonoacetic acid internal standard at +17 ppm.

S1.4c – Calculation of Faradaic Efficiency

The Faradaic efficiency (FE%) of a process, also known as the current efficiency, is defined as the fraction of the total charge passed that proceeds towards a desired reaction product. We assess Q_{PRR} in coulombs for phosphate reduction from the total molar quantity of phosphorus N_{P} collected in the cold trap, bleach trap, reactor head, and transfer lines:

$$Q_{\mathrm{PRR}} = N_{\mathrm{P}} \times \frac{5\,\mathrm{mol\ e^-}}{1\,\mathrm{mol\ P}} \times 96\,485\,\mathrm{C{\cdot}mol^{-1}}.$$

We then normalize this observed value by the gas flow fraction m_{out}:m_{in} (as measured by the flow meters described above) to account for product losses from O-ring leakage, all of which occurs within the cell's working compartment. Meanwhile, Q_{total} is determined from the current integral $\int_0^t i_{total}dt$, which for a galvanostatic experiment is merely $i_{\mathrm{applied}}t$. Putting these values together, we have:

$$\mathrm{FE\%} = \frac{N_{\mathrm{P}} \times \frac{5\,\mathrm{mol\ e^-}}{1\,\mathrm{mol\ P}} \times 96\,485\,\mathrm{C{\cdot}mol^{-1}}}{i_{\mathrm{applied}}t \times \frac{m_{\mathrm{out}}}{m_{\mathrm{in}}}},$$

which for the NMR spectrum depicted in **Figure S12** and its associated galvanostatic bulk electrolysis in **Figure 3** gives us

$$\mathrm{FE\%} = \frac{1.40\,\mathrm{mmol\ P} \times \frac{5\,\mathrm{mol\ e^-}}{1\,\mathrm{mol\ P}} \times 96\,485\,\mathrm{C{\cdot}mol^{-1}}}{75\,\mathrm{mA} \times 13\,878\,\mathrm{s} \times \frac{170\,\mathrm{cm^3{\cdot}min^{-1}}}{250\,\mathrm{cm^3{\cdot}min^{-1}}}}$$

$$= 94.9\%.$$

This value of 94.9% represents a peak observed value for flowthrough-normalized Faradaic efficiency. Preceding attempts at flowthrough-normalized FE% assessment recorded values of 51% (22.3% observed FE out of 45% gas flowthrough) and 76% (41.5% observed FE out of 55% gas flowthrough). We attribute this variability to the ingress of oxygen into the system due to O-ring seal failure at temperature, resulting in the pre-capture oxidation of evolved P_4. Next-generation molten-salt electrolysis reactors may seek to mitigate this factor by incorporating more temperature-resistant gaskets instead of the Kalrez O-rings used in this work.

S1.5 – Experimental Electrochemical Studies

Unless specified otherwise, all electrochemical experiments were conducted with the furnace set to a temperature of 800 °C, which we found to correlate well to the internal equilibrated reactor temperature as measured by an internal thermocouple. Electrochemical data were collected using a Biologic VSP potentiostat using IR compensation values of no more than 20% for noncapacitive voltammetric studies, as higher values were found to induce IR overcompensation at modest current densities. Except where specified otherwise, a graphite electrode was employed as a pseudoreference electrode for all experiments, the potential of which was retroactively corrected for against a Na/Na^+ reference that was briefly (15 seconds – 2 minutes) dipped into the melt before and after each experiment (see above). Uncompensated resistance (R_u) values were measured by the current-interrupt (CI) method; typical R_u values for the melt ranged from 1-10 Ω for all the electrolyte conditions, with higher-melting salts displaying greater solution resistances.

S1.5a – Calculation of Electrochemical Surface Area

Observed currents i were normalized to areal current densities j using electrochemical surface areas (ECSAs) assessed following adapted literature methodologies (*58*). For bare graphite electrodes, specific capacitance values in aqueous solution were found to be approximately 20 $\mu F \cdot cm^{-2}$ relative to the geometric surface area, well in line with literature reports (*59*). Within the melt, specific capacitances of approximately 100 $\mu F \cdot cm^{-2}$ for graphite were determined empirically from geometric surface area. Having determined these values, the ECSAs of an electrode could be determined in terms of the double-layer capacitances (DLCs) of the electrode, both in the melt preceding and following every experiment, as well as within the melt to assess the depth of electrode immersion and ensure the absence of a short between an electrode and the glassy carbon crucible. DLC values were assessed by CV scans spanning ±50 mV around the measured OCP, with three cycles each at scan rates of 10 $mV \cdot s^{-1}$, 20 $mV \cdot s^{-1}$, 30 $mV \cdot s^{-1}$, 40 $mV \cdot s^{-1}$ and 50 $mV \cdot s^{-1}$ at IR compensations of 80%. The resultant capacitive CV traces (**Figure S13**) were used to calculate the total capacitive current i, as the difference of the cathodic current i_c and the anodic current i_a taken at the midpoints (OCP) of the capacitive curve. These currents are related to the overall electrode capacitance C by the relation

$$C = \frac{1}{2} \frac{\partial (i_a - i_c)}{\partial (scanrate)}.$$

Hence, the electrode capacitance (and hence ECSA) could be determined by plotting the capacitive currents as a function of scan rate and dividing the slope of the resulting linear fit, as demonstrated in **Figure S14**.

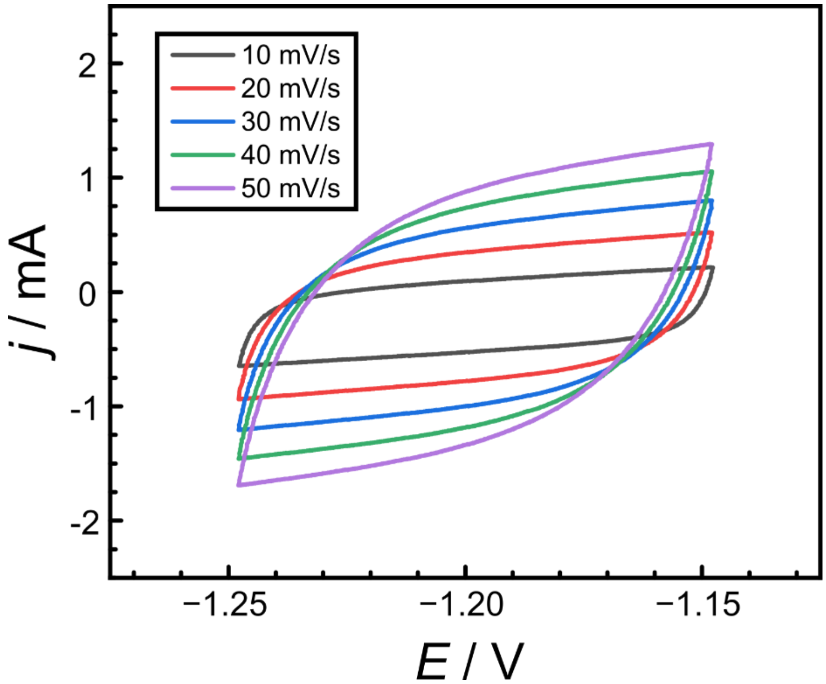

Figure S13. Representative cyclic voltammograms of a graphite electrode scanning ±50 mV around OCP at scanrates of 10 mV·s^{-1}, 20 mV·s^{-1}, 30 mV·s^{-1}, 40 mV·s^{-1} and 50 mV·s^{-1}. Electrolyte is 0.1 M aqueous sodium perchlorate solution.

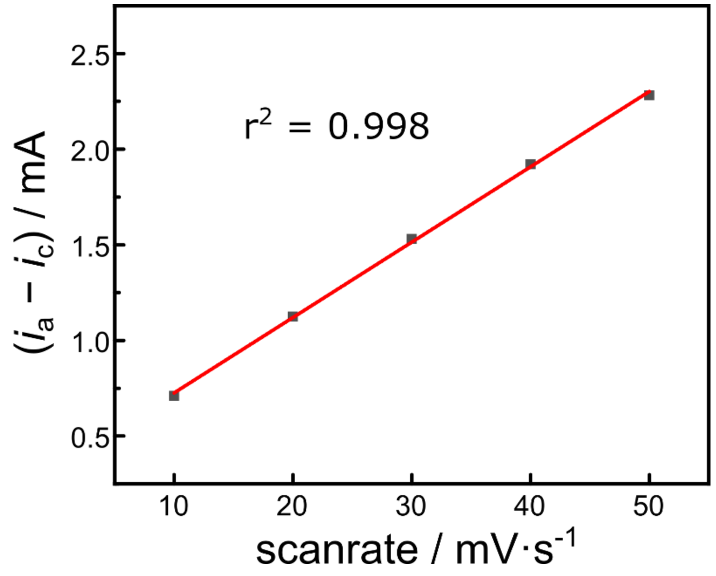

Figure S14. Representative linear fit for scan rate dependence of capacitive current at OCP in **Figure S13**. At an aqueous specific capacitance of 20 μF·cm^{-2}, this slope corresponds to an electrode surface area of 1.08 cm^2.

S1.5b – Collection of Cyclic Voltammetry Data

The collection of cyclic voltammetry data (**Figures 3** and **5**) entailed capacitive CV collection for ECSA determination followed by cyclic voltammetry scans beginning at the open-circuit potential and spanning ±350 mV around the solution OCP, with three scans each at scan rates of 10 $mV{\cdot}s^{-1}$, 25 $mV{\cdot}s^{-1}$, 50 $mV{\cdot}s^{-1}$, and 100 $mV{\cdot}s^{-1}$.

S1.5c – Current-Overpotential Studies

The collection of current-overpotential data for Tafel studies (**Figure 5**) entailed capacitive CV collection for ECSA determination, followed by chronopotentiometric steady state electrolysis. ECSA experiments were performed pre- and post-experiment in both aqueous and molten-salt conditions and found not to substantially differ; however, due to the fixed galvanostatic currents, variation in the electrode surface areas between experiments (due to variable depth of immersion in the melt) is responsible for the slight variation in areal current density values in these plots.

S1.5d – Collection of Galvanostatic Tafel Data

Electrolyses were performed galvanostatically with a fixed current series of 10 µA, 30 µA, 100 µA, 300 µA, 1 mA, 3 mA, 10 mA, 30 mA, 100 mA and 300 mA with 30 second traces and 30 second rest periods at open-circuit between electrolyses. Typically, chronopotentiometry traces reached steady state within 15 seconds. However, for traces in the transition region of the Tafel plots (**Figure 5**), the potential decayed to more negative values over the course of 30 seconds before reaching steady state. This slower approach to steady state is attributed to anhydride depletion local to the electrode. In all cases, the final potential at the conclusion of chronopotentiometry trace was taken as the steady-state value plotted in **Figure 5**. Voltage compensation for solution resistance was implemented manually during data processing. Each galvanostatic series was performed sequentially in triplicate in every experimental setup, and the experimental setups themselves were replicated between three and nine times each to account for random variations in reactor arrangement. Error bars were calculated from 95% confidence intervals as 1.95996 times the sample standard error.

S1.5e – Determination of Overpotential

Overpotentials for phosphate reduction were determined for each melt in this study relative to E_{eq} values determined by the hollowed-electrode method. In particular, a hollowed electrode was immersed in each melt, and gently agitated to dislodge air bubbles trapped in the electrode hollow. P_4 was generated in the electrode hollow by reductive galvanostatic electrolysis at 10 $mA{\cdot}cm^{-2}$ for one minute and then a 5-minute open-circuit trace was recorded (**Figure S17**). E_{eq} was taken to be the minimum recorded OCP over this 5-minute trace, discounting double-layer relaxation immediately following electrolysis. This methodology was further vetted by post-galvanostatic anodic linear-sweep voltammetry (LSV) allowing a five-second post-electrolysis rest at OCP (**Figure S15**), as well as both analogous LSV and OCP studies on a red phosphorus-dosed hollow graphite electrode (**Figure S16**).

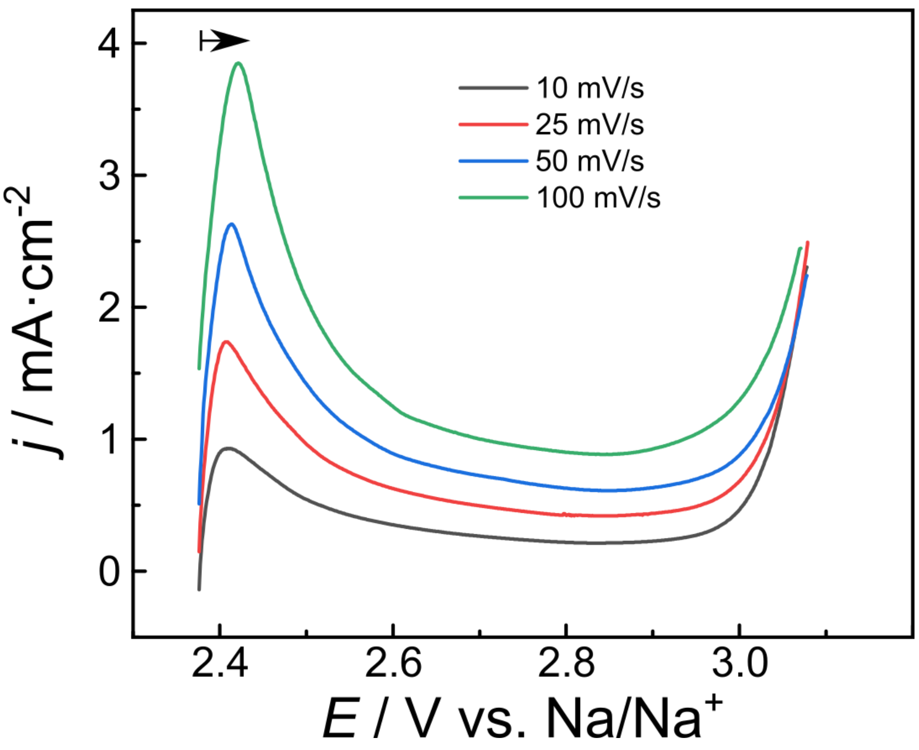

Figure S15. LSV traces of a hollowed graphite electrode following a 1-minute galvanostatic electrolysis at −10 mA·cm^{-2}, scanning oxidatively from OCP at scan rates of 10 mV·s^{-1}, 25 mV·s^{-1}, 50 mV·s^{-1}, and 100 mV·s^{-1}.

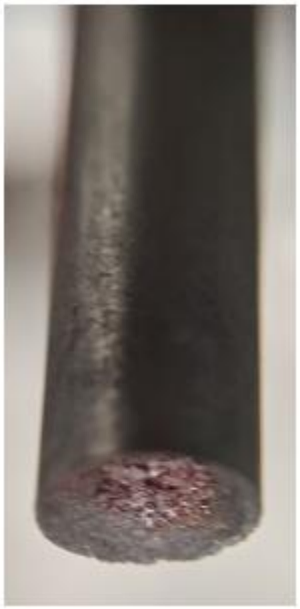

Figure S16. A hollowed graphite working electrode, with the produced cavity smeared with red phosphorus for non-Faradaic *in situ* P_4 generation.

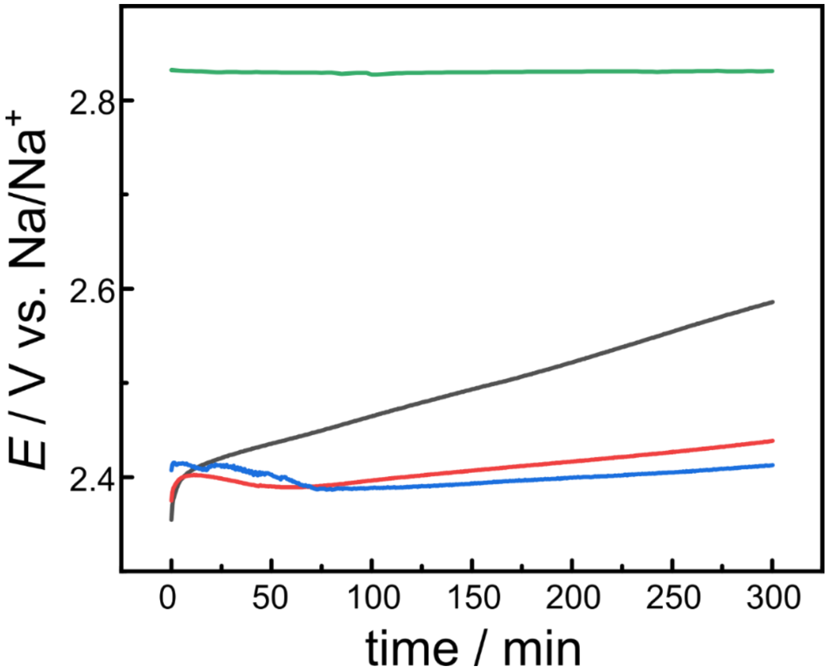

Figure S17. 5-minute OCP traces of a resting (pre-electrolysis) sharpened graphite electrode (green), sharpened (black) and hollowed (red) graphite electrodes following 1-minute galvanostatic electrolyses at −10 mA·cm^{-2}, and a hollowed graphite electrode coated in red phosphorus (blue).

S1.5f – Methodology for Tafel Analysis

The relationship between the cathodic current density j and the overpotential η is defined by the Tafel equation:

$$\eta = -\frac{RT \ln 10}{\beta n F} \log_{10}\left[\frac{j}{j_0}\right],$$

where β is the symmetry factor for a single-step electrochemical reaction in which n electrons are transferred and j_0 is the exchange-current density. The symmetry factor is defined as a number between 0 and 1, corresponding to the relative position of the activated complex as a fraction of the reaction coordinate from reduced to oxidized species. Experimentally determined symmetry factors typically range from 0.4 to 0.6, and hence β is typically assumed to be 0.5 in nearly all cases (*41*). Meanwhile, the exchange-current density is defined as the equilibrium current density at net zero overpotential, at which the magnitudes of anodic and cathodic current are equal, thereby evincing no net electrolysis.

Generalizing the Tafel equation to a multistep case, we have

$$\eta = -\frac{RT \ln 10}{\alpha F} \log_{10}\left[\frac{j}{j_0}\right],$$

in which the single-step symmetry factor-adapted electron transfer number $n\beta$ is replaced by the multi-step *transfer coefficient*

$$\alpha = \frac{\gamma}{\nu} + r\beta,$$

where γ is the number of electrons transferred prior to the rate-limiting step (RLS), ν is the stoichiometric number of the reaction, r is the number of electrons transferred in the RLS, and β is the symmetry factor of the RLS (*41–43*). At 800 °C, this corresponds to a Tafel slope

$$b_{800\ ^\circ\mathrm{C}} = \frac{\partial\eta}{\partial(\log j)} = \frac{212.9\,\mathrm{mV{\cdot}dec^{-1}}}{\alpha}.$$

Tafel slopes and errors were assessed by least-squares linear fitting (using OriginLab Origin2018's Linear Fit with Y Error algorithm to propagate errors) of the current-potential data in the 'macropolarization' regime, defined as current densities ≥1 $\mathrm{mA{\cdot}cm^{-2}}$. All Tafel slopes are reported in units of $\mathrm{mV{\cdot}dec^{-1}}$.

S1.6 – Gas Chromatography of Gas Outflow Streams

For analysis of counter electrode outflow streams, GC analysis was performed by injection of the effluent gas into a Multi-Gas Analyzer (#3; SRI Instruments) equipped with a thermal conductivity detector, methanizer, and flame ionization detector in series after Molsieve 13x and Hayesep D Columns. With the counter compartment gas flowrate set to 60 sccm, baseline measurements were collected by injecting gas from the counter electrode outflow stream at rest, while experimental values were collected by injecting gas from the counter electrode outflow stream while passing a fixed cathodic current of 75 mA through the working electrode (and thereby an equivalent anodic current through the counter electrode).

The GC traces evince a significant increase in the gas fractions of CO and CO_2 while the counter electrode is under anodic polarization relative to a control experiment without electrolysis. Integrating the GC traces reveals a gas outflow stream that is 96% CO_2 and 4% CO, equivalent to a net electron efficiency of 3.9 electrons per atom of carbon, out of a theoretical 4 electrons per carbon for the total conversion of $C \rightarrow CO_2$. This compares favorably to the value of 2 electrons per carbon for the incomplete combustion $C \rightarrow CO$ used in the thermal process. The generation of CO may be attributable to either the partial oxidation of graphite and/or the Boudouard reaction, the comproportionation of CO_2 with carbon, $CO_2 + C \rightleftharpoons 2\ CO$ (*60-62*).

S1.7 – Composition and Structure of Molten Condensed Phosphates

Condensed phosphates are defined as phosphate salts that are dehydrated relative to free orthophosphate, possessing at least one phosphoryl anhydride linkage between adjacent phosphorus atoms. Collectively, the condensed phosphates comprise the linear polyphosphates, the cyclic metaphosphates, and the branched ultraphosphates (**Figure S18**). These phosphoryl anhydride linkages are crucial to describing the chemistry of the condensed phosphates, as their oxide-induced cleavage to terminal phosphates is fundamental to the 'oxide-accepting' character of condensed phosphate melts (**Figure S19**).

This reactivity allows condensed phosphate melts to intrinsically fulfill the role performed in the thermal process by the conversion of silicon dioxide to metasilicate, which is associated with a majority of the slag formation (and associated energetic losses) of the thermal process.

Individual condensed phosphate species consist of a mixture of terminal, branching, and bridging moieties such that, by the relations detailed in **Table S5**, all condensed phosphate ions will satisfy the relation $[(PO_{2.5})_u(PO_3)_m(PO_{3.5})_p]^{(m+2p)-}$, where u is the number of branching (ultraphosphate) moieties, m is the number of bridging (metaphosphate) moieties, and p is the number of terminal (paraphosphate) moieties. While the presence of fractional oxygen atoms for bridging and terminal phosphate groups may seem problematic at first, the nature of terminal and branching groups in a phosphate oligomer is such that the total number of bridging and terminal phosphates must always be an even number, so this is not an issue. For obvious reasons, free orthophosphate ions cannot be part of a condensed phosphate oligomer, as they have no phosphoryl anhydride linkages and hence are not condensed phosphates. The interconversion of phosphate oligomers – for example, of two paraphosphates into an orthophosphate and a metaphosphate, two metaphosphates into a paraphosphates and an ultraphosphate, or the reverse of these reactions – is expected to occur rapidly at the high temperature of the melt and, thus, we consider all molten phosphate species to be in equilibrium under the conditions of electrolysis (*63*). As the precise distribution of condensed phosphate chains is a function of temperature, precise characterization of a melt cannot be performed by analysis of the solidified electrolyte, although such post-solidification analysis vindicates our conflation of mixed orthophosphate-metaphosphate solutions with their equivalent pure polyphosphate melts (see below). Future studies on the *in situ* character of the melt (perhaps by high-temperature NMR) may permit quantification of the phosphate speciation in the reaction media.

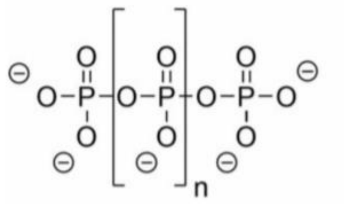

(a) Structure of a polyphosphate ion, $n \geq 0$.

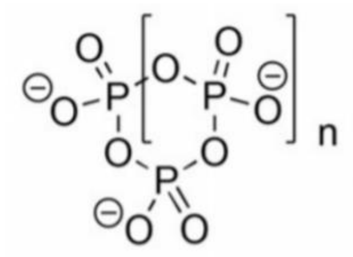

(b) Structure of a metaphosphate ion, $n \geq 1$.

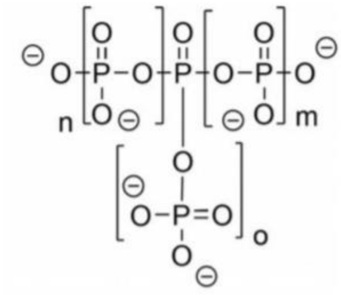

(c) Structure of an ultraphosphate ion, $m, n, o \geq 1$.

Figure S18. Classifications of condensed phosphate species.

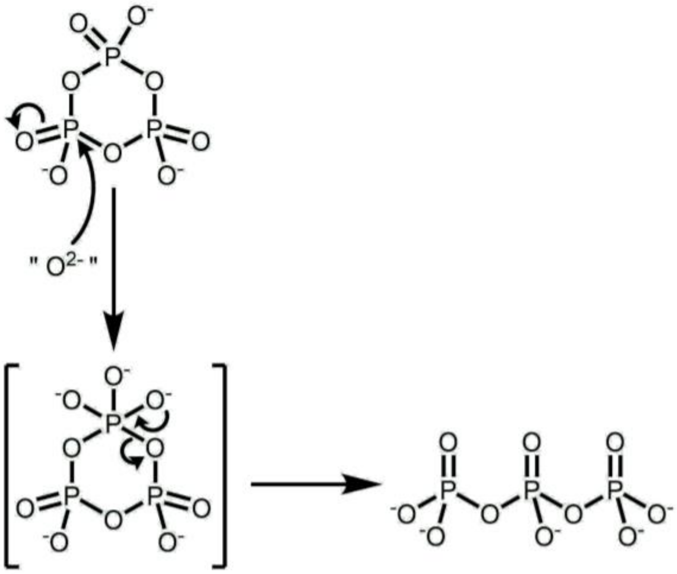

Figure S19. Representative mechanism for cleavage of a phosphoryl anhydride linkage by an oxide equivalent, here showing the formation of tripolyphosphate ($P_3O_{10}^{5-}$) from trimetaphosphate ($P_3O_9^{3-}$). Note that oxide equivalents rarely exist as free O^{2-} ions, and in practice are more likely to be represented as nucleophilic polyphosphate chains.

Table S5. Phosphate species by the number of phosphoryl anhydride linkages per phosphorus atom and their equivalent oxide content relative to phosphoric anhydride, P_4O_{10}.

Prefix	Structure	Anhydrides	Oxides	Formula Unit
ortho-	free	0	1½	PO_4^{3-}
para-	terminal	1	1	$PO_{3.5}^{2-}$
meta-	bridging	2	½	PO_3^{-}
ultra-	branching	3	0	$PO_{2.5}$

S1.7a – Preparation of Selected Condensed Phosphate Mixtures

As seen in **Table S6**, four selected condensed phosphate melts of varying degrees of oxide content were selected for analysis in this work, spanning a range of phosphoryl anhydride molalities from 5.4 $mol \cdot kg^{-1}$ to 9.8 $mol \cdot kg^{-1}$. While the first of these melts was equivalent to a pure metaphosphate melt, the remainder were synthesized by mixing targeted mole fractions of sodium trimetaphosphate (S3MP) and sodium orthophosphate (SOP) to produce a homogenous electrolyte upon fusion. A 75:25 mol:mol mixture of S3MP:SOP (85:15 by mass) is 8.3 $mol \cdot kg^{-1}$ in phosphoryl anhydride linkages, equivalent to that of sodium decapolyphosphate (S10PP); a 50:50 mol:mol mixture (65:35 by mass) is 6.4 $mol \cdot kg^{-1}$, equivalent to sodium tetrapolyphosphate (S4PP); finally, a 40:60 mol:mol mixture (55:45 by mass) is 5.4 $mol \cdot kg^{-1}$, equivalent to sodium tripolyphosphate (S3PP). Lower concentrations of phosphoryl anhydride linkages are largely inaccessible as melts at the reaction temperature of 800 °C; the next lowest polyphosphate, sodium pyrophosphate (3.8 $mol \cdot kg^{-1}$ in phosphoryl anhydride linkages) has a melting point of 988 °C, and pure sodium orthophosphate (0 $mol \cdot kg^{-1}$ in phosphoryl anhydride linkages) has a melting point of 1583 °C. For each of these selected melts, a total mass of 60 g was measured out in the prescribed ratio of S3MP (99.9999%, anhydrous, #AA89063A1 from Alfa Aesar™) to SOP (99.9999%, anhydrous, #AC389810010 from Acros Organics™) and ground together in a mortar & pestle to ensure homogeneity. This mixture was then poured into a glassy carbon crucible for electroanalysis as described above.

Table S6. Condensed phosphate melts selected for study, their net anhydride content (in mol·kg^{-1}), their homogeneous equivalent species (in terms of melt oxide/anhydride content), and the molar ratios of sodium trimetaphosphate (S3MP) and sodium orthophosphate (SOP) required for synthesis.

**Melt Equivalent**	**[anhydride]**	**$Na_3P_3O_9$ mol%**	**Na_3PO_4 mol%**
S3MP ($Na_3P_3O_9$)	9.8076	100%	0%
S10PP ($Na_{12}P_{10}O_{31}$)	8.3210	75%	25%
S4PP ($Na_6P_4O_{13}$)	6.3883	50%	50%
S3PP ($Na_5P_3O_{10}$)	5.4368	40%	60%

Section S2 – Supplemental Experiments

S2.1 – Replenishment of Anhydride-Depleted Melt with Phosphoric Acid

S2.1a – Electrochemical Characterization of Anhydride-Depleted Polyphosphate Melt

In order to simulate a melt with a depleted concentration of anhydride bonds after extended electrolysis, 18.1420 g (0.110 mol) of Na_3PO_4 was combined with 33.8582 g (0.110 mol) of $Na_3(PO_3)_3$ to produce 52 g of a 50 mol % Na_3PO_4 / 50 mol% $Na_3(PO_3)_3$ mixture, with an expected anhydride molality of 6.4 $mol{\cdot}kg^{-1}$. This mixture was heated and melted in the molten salt electrochemical reactor, and the melt was subsequently characterized via cyclic voltammetry at 800 °C (**Figure S20,** "Anhydride-Depleted Electrolyte") with a sharpened graphite working electrode whose surface area was previously measured via capacitance.

S2.1b – Phosphoric Acid Addition and Dehydration

After characterization and cooling, the phosphate salt compact was removed from the crucible and ground into a fine powder with a mortar and pestle. 0.086 g of this powder was saved for NMR analysis (see below), and 39.94 g (0.085 mol tetrapolyphosphate on average) of the powdered mixture was mixed with 19.60 g (0.17 mol) of 85 wt % phosphoric acid, the stoichiometric amount to permit complete conversion of the melt composition into $Na_3(PO_3)_3$ after dehydration. This mixture was placed in the glassy carbon crucible, left to set under a flow of nitrogen overnight, and then reheated via a slow manual ramp from room temperature up to 800 °C, where it was held at temperature overnight and then cooled. This melt is referred to as the phosphoric acid treated melt.

S2.1c – Electrochemical Characterization Following Addition of Phosphoric Acid

After cooling, the melt compact was again ground into a powder via mortar-and-pestle. While the 50%/50% mixture had been a semi-crystalline solid that was difficult to pulverize, the new melt compact was a soft, crumbly solid that readily broke apart, indicating compositional changes. Roughly 0.1 g of the phosphoric acid-treated melt was saved for NMR analysis (see below) and the remainder was re-melted in the molten salt electrochemical reactor. The melt was subsequently characterized via cyclic voltammetry at 800 °C on the same sharpened graphite working electrode (**Figure S20**, "Replenished Electrolyte"), whose surface area was previously evaluated via capacitance measurements to have a value of 13.6 cm^2. The current densities reported were corrected from raw current using this value, in the manner of **Section S1.5a**.

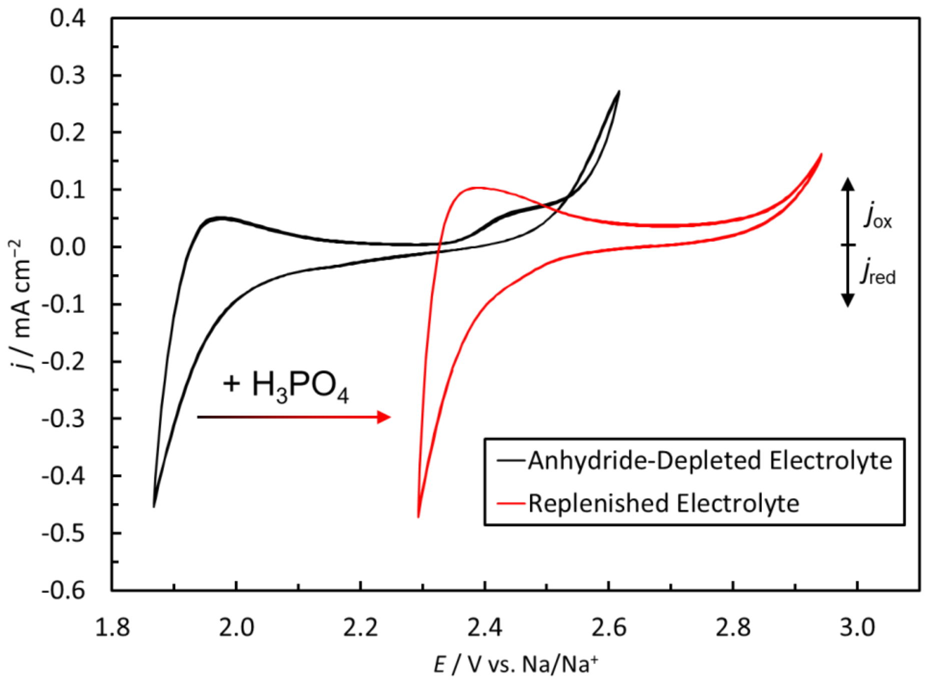

Figure S20. Cyclic voltammetry (black) of a sodium polyphosphate melt containing an anhydride molality of 6.4 mol·kg^{-1}. This low anhydride molality simulates the electrolyte composition following extensive electrolysis. Cyclic voltammetry (red) following addition of phosphoric acid to the low anhydride melt to generate an anhydride molality of 9.2 mol·kg^{-1}. Data were recorded at 100 mV·s^{-1} scan rate at 800 °C on a sharpened graphite rod working electrode. Potentials are reported vs. the Na/Na^{+} redox couple.

S2.1d – ^{31}P NMR Characterization Prior to and Following Phosphoric Acid Addition

The powder samples of the anhydride-depleted and phosphoric acid-treated melts were dissolved in 0.6 mL of deuterated water and characterized by ^{31}P NMR (**Figure S21)**. ^{31}P NMR spectra for the anhydride-depleted melt display resonances at −5 and −20-21 ppm corresponding to polyphosphate 'end' groups (-O-PO_3^{2-}) and polyphosphate 'middle' groups (-O-PO_2^{-}-O-), respectively. Relative integrations of these peak families indicate an 'end' to 'middle' ratio of approximately 1:1, which would be consistent with an average composition corresponding to sodium tetrapolyphosphate. In contrast, the phosphoric-acid treated melt displays a single resonance at −21 ppm corresponding to pure sodium trimetaphosphate. This indicates that phosphoric acid treatment and dehydration is sufficient to regenerate the anhydride bonds in the native melt and allow for continued replenishment of the electrolyte.

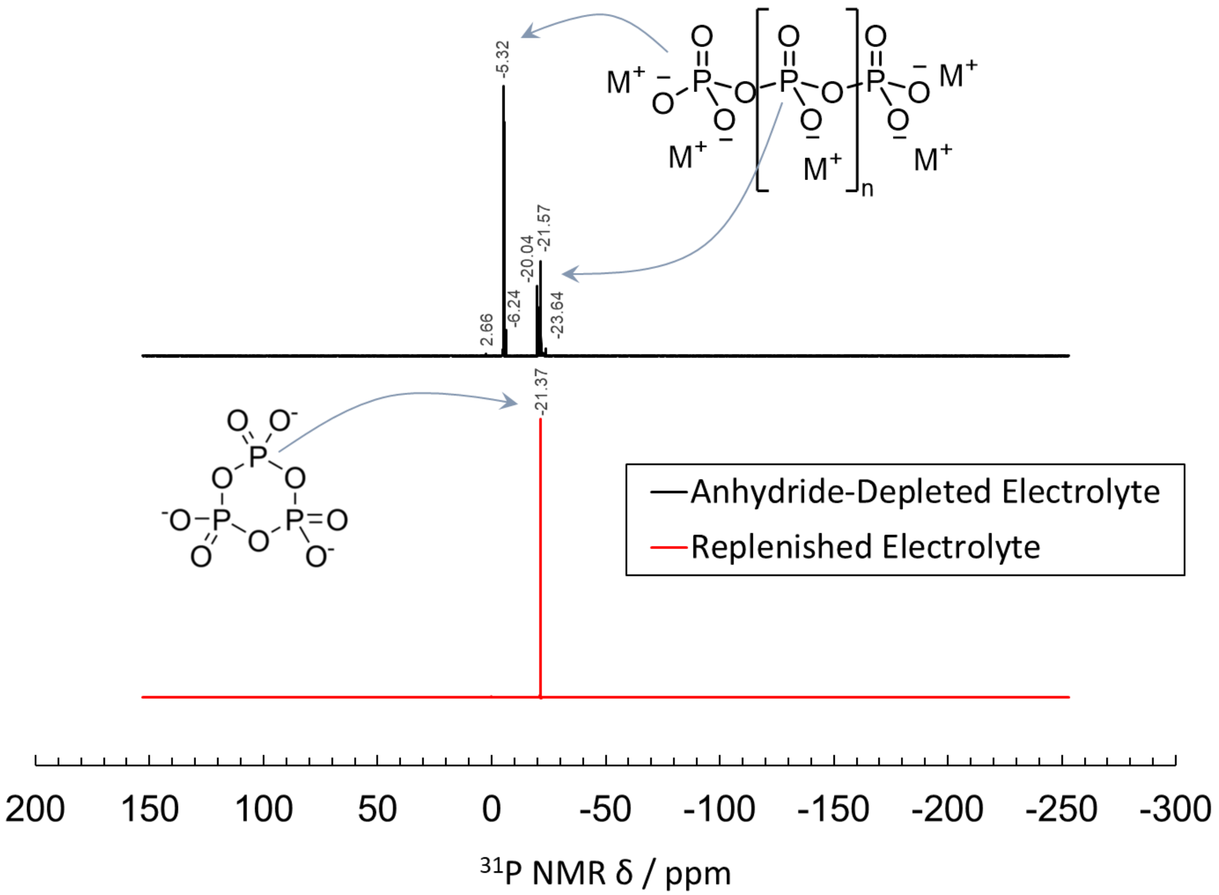

Figure S21. ^{31}P NMR of the anhydride-depleted (black) and phosphoric acid-replenished (red) samples in D_2O. Arrows indicate assignments of the peak families to phosphorus atoms in the respective polyphosphate structures.

S2.2 – Electrolysis of "Impure" Phosphoric Acid

Wet-process phosphoric acid contains an array of impurities. Thus, we investigated the electrochemical behavior of a melt generated from impure phosphoric acid. To simulate an impure polyphosphate melt, we combined the following listed compounds (**Table S7**), in their respective proportions known to exist in wet-process phosphoric acid (*64*). This mixture was added to a glassy carbon crucible and heated to 800 °C in the electrochemical furnace. This heating served to both dehydrate and melt the impure polyphosphate mixture. Electrochemical measurements were then performed with graphite rod electrodes using the procedures described above. Despite the large population of impurity ions, the voltammetric signatures resemble those of a pure polyphosphate melt with a phosphoryl anhydride molality of 6.4 $mol \cdot kg^{-1}$ (**Figure S22**). This data suggests that the presence of impurities does not dramatically impede the native electrochemical processes for P_4 synthesis.

Following prolonged electrolysis for 2 hrs at a current density of 12.8 $mA \cdot cm^{-2}$ on a graphite cone electrode (corrected by the submerged surface area of 7.84 cm^2, measured via capacitance), the cell was allowed to cool to room temperature and was subsequently disassembled. The graphite cathode was coated around the tip with a black powdery deposit which we attribute to the reduction of impurity metals in the melt and/or to the reaction of the produced P_4 with metal impurities to form metal-phosphide phases. Given the voltammetric similarity between the pure and impure melts, we favor the latter explanation. Irrespective of the mechanism, these findings suggest that impurity induced the formation of insoluble deposits on the electrode, facilitating their periodic removal.

Table S7. Chemical constituents for a melt simulating sodium metaphosphate derived from 'dirty' phosphoric acid at industrial wet process concentrations.

	**Wt% in wet-process phosphoric acid**	**Analog Species**	**Amount Used**
H_3PO_4	75.3	**H_3PO_4 85 wt %**	9.3921 g
		**$Na_3(PO_3)_3$**	43.6935 g
P_2O_5	54.6		
CaO	0.15	**$CaSO_4$*$2H_2O$**	0.3062 g
MgO	1.09	**$Mg_5(CO_3)_4(OH)_2$*$3H_2O$**	1.6136 g
Al_2O_3	0.86	**Al_2O_3**	0.5707 g
Fe_2O_3	0.48	**$FePO_4$*H_2O**	0.6740 g
SO_4^{2-}	1.03	**Na_2SO_4**	0.7598 g
F^-	0.048	**F-**	-
C	0.03	**C (carbon black)**	0.0200 g

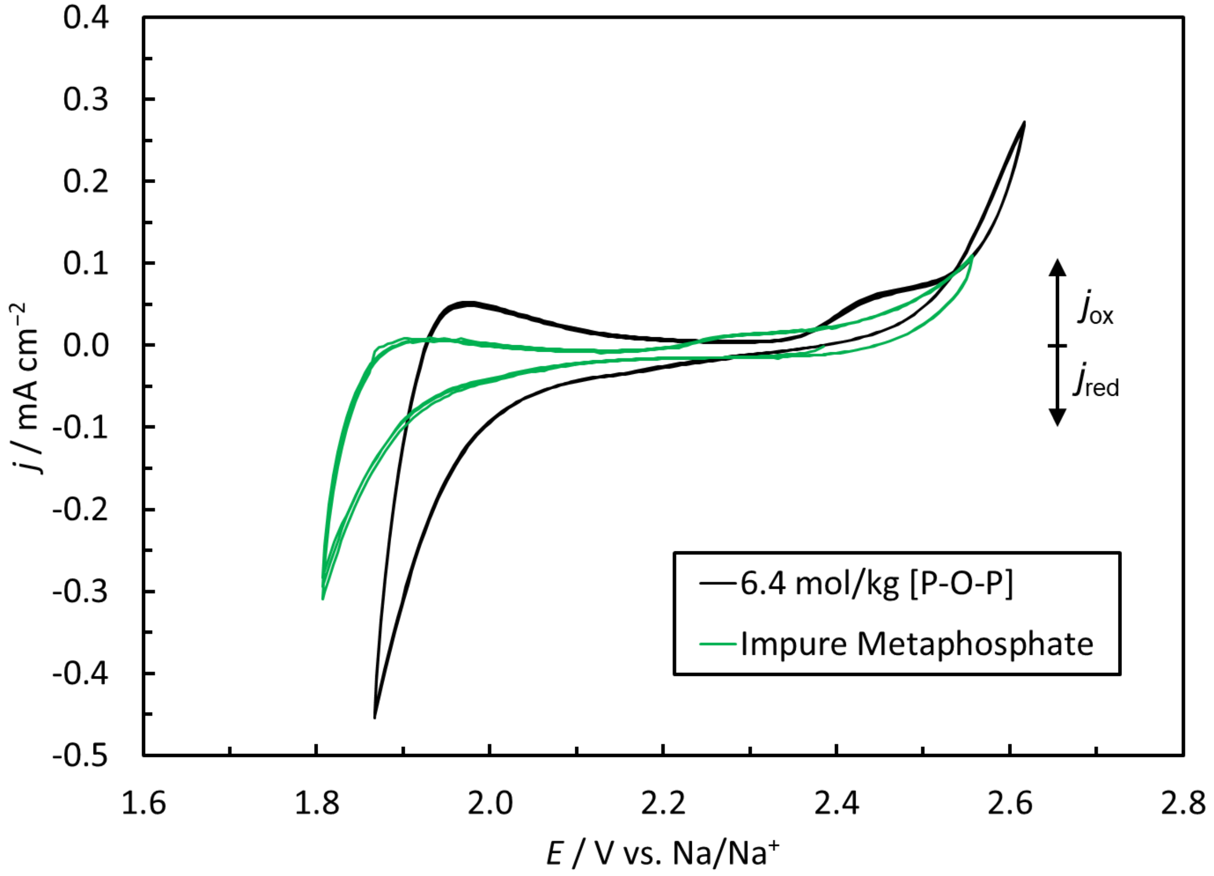

Figure S22. Cyclic voltammetry of a metaphosphate solution prepared from the impurities in dirty phosphoric acid (green), overlaid with cyclic voltammetry of a typical 6.4 mol·kg^{-1} melt of sodium polyphosphate (black). Data were recorded on a sharpened graphite rod work electrode. Potential are reported vs the Na/Na^+ redox couple.

S2.3 – Solubility of Calcium in Condensed Phosphate Melts

Tuning the anhydride population provides an additional handle for controlling the solubility and managing the build-up of impurity ions.

Prior studies establish that pure metaphosphate metals are able to solubilize substantial quantities of Ca^{2+} ions. Indeed, Na^{+} and Ca^{2+} metaphosphate binary melts remains liquid at the 800 °C operating temperature of our system up to a 49 mass % of calcium metaphosphate in sodium metaphosphate (0.33 mole fraction of Ca^{2+}) (*35*). The solid phase in equilibrium with the melt is reported to be enriched in, if not entirely comprised of, calcium metaphosphates. This data suggests that Ca^{2+} impurities would remain quite soluble in pure metaphosphate melts.

To best of our knowledge, no studies have examined Ca^{2+} solubility as a function of anhydride concentration in polyphosphate melts. As a qualitative assessment, we examined whether various ratios of calcium orthophosphate ($Ca_3(PO_4)_2$) and sodium trimetaphosphate would form precipitates at the operation temperature of 800 °C. Powders of $Na_3(PO_3)_3$ and ($Ca_3(PO_4)_2$) were mixed in the ratios in **Table S8**. Each sample was transferred to a quartz crucible and heated to 800 °C. They were held at this temperature overnight to provide adequate time to equilibrate, and then visually inspected.

At the high anhydride molality of 8.9 $mol{\cdot}kg^{-1}$, the presence of 6.7% Ca^{2+} still leads to a transparent melt (**Figure S23a**). In contrast, a cloudy melt containing visible precipitates is observed for a 14% Ca^{2+} fraction at a slightly lower anhydride molality of 7.7 $mol{\cdot}kg^{-1}$ (**Figure S23b**). Higher Ca^{2+} percentages and lower anhydride molalities lead to solid compacts or powders at 800 °C (**Table S8**). This qualitative behavior contrasts with the ability of metaphosphate melts (anhydride molality ~9.8 $mol{\cdot}kg^{-1}$) to support 33% Ca^{2+} and highlights that anhydride molality can be used to tune the soluble of divalent ions in the melt.

Table S8. Prepared mixtures of calcium orthophosphate and sodium trimetaphosphate and their physical states after being held overnight at 800 °C. Melt compositions represented both by mole percentage of calcium ions relative to sodium ions in the melt, as well as theoretical anhydride bond molality in the melt, in mol·kg^{-1}.

**Calcium Phosphate (g)**	**Sodium Trimetaphosphate (g)**	**Mole % Ca^{2+} (in Na^+)**	**Theoretical anhydride molality (mol·kg^{-1})**	**State at 800 °C**
1.04	8.95	6.7%	8.9	Uniform liquid
2.15	7.85	14%	7.7	Liquid with white precipitate
5.51	4.48	33%	4.4	Solid compact
7.00	3.00	59%	2.9	Crumbly cake of powders

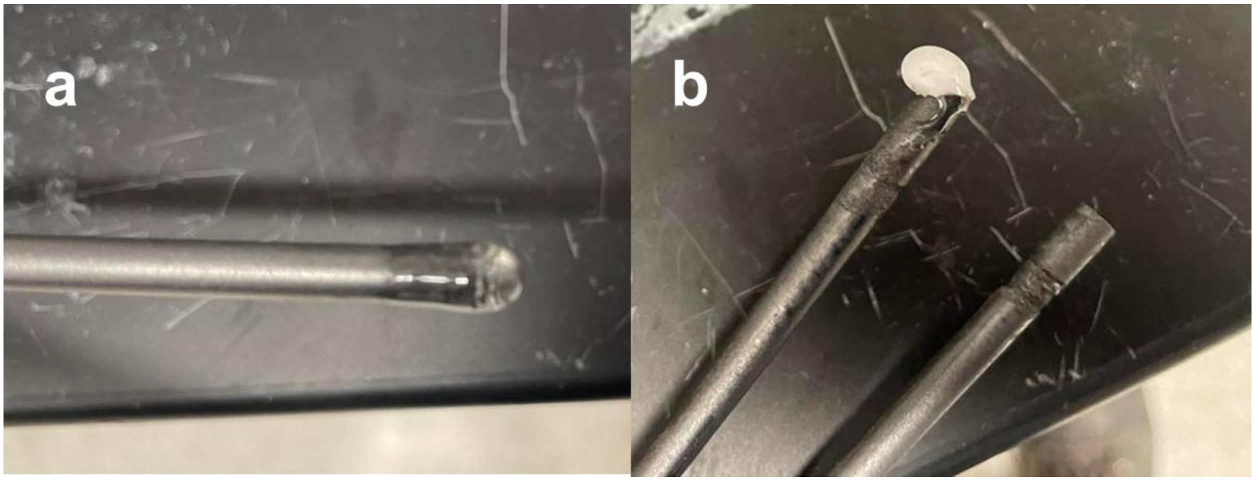

Figure S23. Photographs of select melts tested for precipitation of a calcium ion-based solute. a) Aliquot of a melt containing 6.7% Ca^{2+} and an anhydride molality of 8.9 mol·kg^{-1} showing a clear glassy appearance with no precipitate. b) Aliquot of a melt containing 14% Ca^{2+} and an anhydride molality of 7.7 mol·kg^{-1} showing a cloudy appearance with visible precipitates. Photographs were taken immediately upon removal of the aliquot from the 800 °C melt.

S2.4 – Longer-Term Electrolysis

Typically, electrolyses were manually terminated after 30 min to 3 hours so as not to generate excessive amounts of white phosphorus. However, we have conducted several long-term electrolyses, and representative 17-hour electrolysis trace at a -30.4 mA·cm^{-2} current density (corrected via capacitive area of 9.87 cm^2) is shown in **Figure S24**. Running the reaction beyond this point is a challenge given the limits of our experimental design for several engineering reasons, including:

a) the neck leading to our collection flask is narrow and eventually clogs with condensed red phosphorus, preventing further nitrogen flow,

b) our research-grade graphite electrodes are thin and fragment over time due to anodic corrosion; we have no means to add more graphite *in situ* as the reaction progresses.

c) we employ no liquid-phase separator or membrane in our small-scale system and, thus, crossover of loose graphite particles released from aggressive anode oxidation can short the electrochemical cell. The potential spike in **Figure S24** is attributed to this phenomenon.

d) because we are operating with a fixed electrolyte volume, and do no replenish it *in situ* via the methods we discuss earlier (see "Replenishment of Anhydride-Depleted Melt with Phosphoric Acid" and **Figure S22**), the Lux acidity of the system is depleted over time and this can a progressive drift in the working electrode potential.

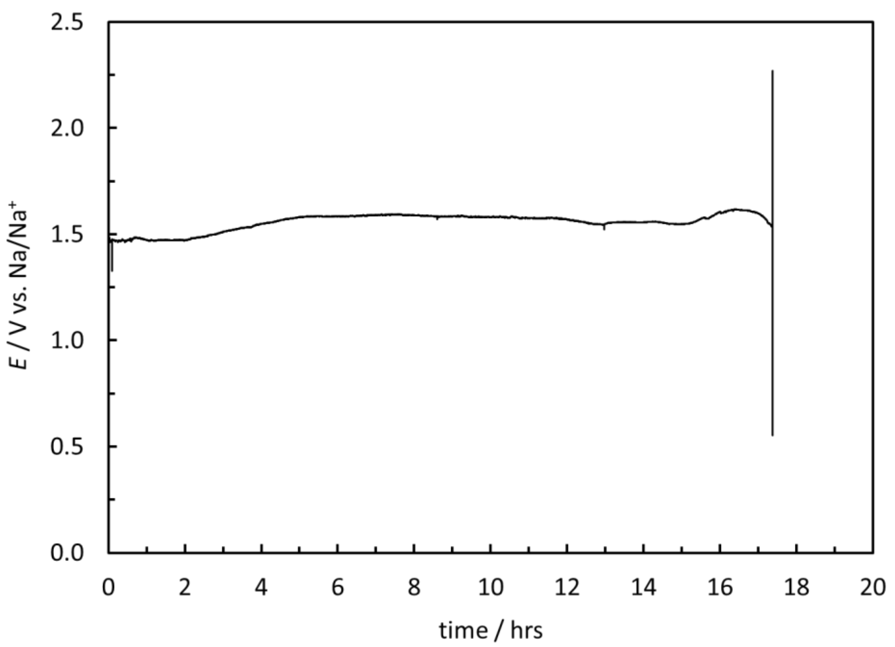

Figure S24. Long-term chronopotentiometry data at 30.4 mA·cm^{-2} on a sharpened graphite working electrodes in a molten sodium metaphosphate melt at 800 °C. The abrupt spike in potential at ~17 hrs is attributed to the progressive corrosion of the graphite anode and the formation of graphite particle in the melt that eventually short the electrochemical cell.

S2.5 – Steady-State Kinetics of Graphite Rod Oxidation

In order to examine whether the kinetic benefits of high Lux acidity for PRR are mitigated by attenuation kinetics at the carbon anode, we collected steady-state chronopotentiometry of the graphite oxidation reaction at the endpoint of high and low Lux acidity examined in this study. Specifically, the anhydride concentrations were 9.8 mol kg^{-1} (pure metaphosphate) and 5.4 mol kg^{-1} (produced from 23.1743 g Na_3PO_4 and 28.8263 g $Na_3(PO_3)_3$). Constant current electrolyses were performed at 800 °C and the steady-state potentials were measured relative to a graphite pseudoreference and Na/Na+ primary reference as detailed above. Electrode surface areas was evaluated via capacitance of the submerged graphite tip in the melts using the 100 μF cm^{-2} specific capacitance value determined in Section S1.5a. Steady-state voltage values were measured at for electrolyses conducted over a range of currents spanning 10 μA to 200 mA and the resulting Tafel data is plotted in **Fig. S25**. Before collection of each chronopotentiometry data point, the solution resistance was measured and used to correct the observed steady state potential for ohmic losses.

The top plot in **Fig. S25** shows the polarization data referenced a Lux-acidity independent Na/Na^+ reference and shows the offset between the two curves caused by shifts in the thermodynamic potential for carbon oxidation. The bottom plot in **Fig. S25** references the data to a graphite pseudoreference which we found to closely pin to the phosphate/P_4 equilibrium potential. Thus, the bottom plot accounts for shifts in the equilibrium potential with changing Lux acidity and captures differences in overpotential between the two melts. While a greater overpotential penalty is observed for the high Lux acidity melt (**Fig. S25** black), the sensitivity to Lux acidity is less pronounced than for PRR. At 30 mA cm^{-2} current density, we observe ~0.6 V increase in overpotential upon going from low to high Lux acidity. In contrast, at the same current density, we observed a ~1.1 V reduction in overpotential for P4 production in the high Lux acidity melt (**Figure 5**). Thus, this data suggests a ~0.5 V reduction in overall cell voltage at this current density for the high Lux acidity melt.

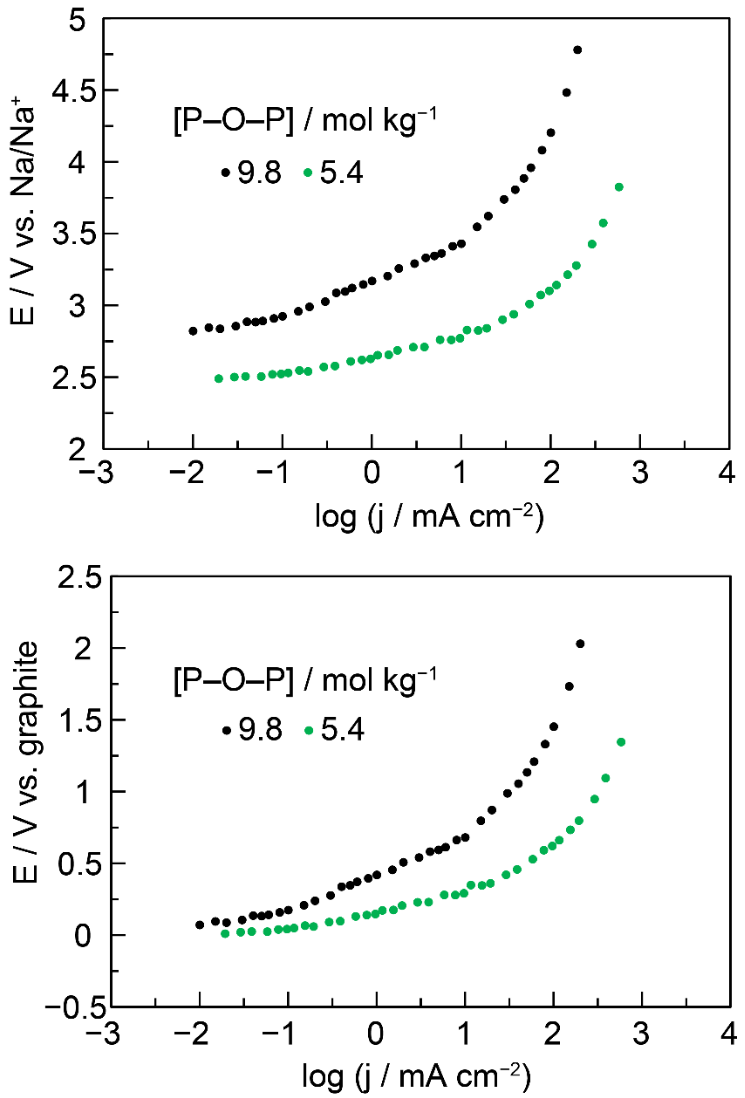

Figure S25. (*Top*) Steady-state voltage for oxidation of graphite vs. a Na/Na^+ reference electrode with respect to chronopotentiometric current density in both sodium trimetaphosphate (black) and sodium tripolyphosphate (green) electrolyte melts. (*Bottom*) Same data as top, with voltages expressed relative to the graphite pseudoreference potential which accounts for shifts in the equilibrium potential with changing Lux acidity.

S2.6 – XPS of Graphite Cathode Tips

In order to investigate local species generated at the graphite cathode, X-ray photoelectron spectra (XPS) were collected on the tips of three graphite cathodes. Two cathodes were prepared using the methods described in section S1.1c, while the third was additionally oxidized with aqua regia before use as a cathode in order to interrogate whether the oxidation state of the surface affected its electrochemical properties. After preparation, each of these graphite rods were individually utilized in the melt as cathodes for the steady-state analysis of phosphorus evolution kinetics, with current densities ranging from 10 μA cm^{-2} to 100 mA cm^{-2}. After use, these electrodes were subsequently removed from the melt, cooled and their tips severed from the graphite rod and examined under XPS.

X-ray photoelectron spectra of these graphite electrode tips (**Fig. S26**) were recorded using a Physical Electronics PHI Versaprobe II with a monochromatic aluminum Kα X-ray source (1486.6 eV) and a hemispherical energy analyzer. Spectra were collected by fixing the graphite rod tips to the support platen with conductive carbon tape. Data were collected at a base pressure of 5×10^{-9} torr using a 200 μm, 50 W focused beam at a take-off angle of 45°. Survey spectra (**Fig. S26a**) were collected using a pass energy of 187.85 eV and a step size of 0.8 eV. High energy resolution scans that were used for peak fitting (**Fig. S26b-d**) were collected with a pass energy of 23.50 eV and a step size of 0.1 eV. Element identification was performed using MultiPak software (version 9.6.3.B).

The only elements consistently observed in all three samples (**Fig. S26a**) were carbon, sodium, oxygen, and phosphorus, as was anticipated for a graphite electrode in contact with a sodium metaphosphate melt. In one of the two electrodes prepared without aqua regia, a small silicon peak was also observed, which was attributed to accidental contact of that particular sample with the crucible furnace's ceramic foam cap. Peak splitting in the phosphorus and oxygen regions (**Fig. S26c-d**) were observed for all samples, which was attributed to differential charging during spectroscopic analysis due to the nonconductive nature of the salt films. When an argon neutralizer gun was employed, the splitting behavior vanished.

In all samples, the carbon 1s profile (**Fig. S26b**) showed a single peak at 284.5 eV consistent with graphite, indicating that the surface had not significantly changed as a result of the electrochemistry or, in the case of the aqua regia-treated sample, the oxidative pretreatment. Under the neutralizer gun, the oxygen 1s region (**Fig. S26c**) displayed a single peak at 532.5 eV consistent with a phosphate-bound oxide, while the phosphorus spectrum (**Fig. S26d**) showed a single peak at 135.5 eV consistent with P(V) phosphate species. In contrast, reduced phosphorus species are expected to display peaks in the 133-131 eV binding energy range. No peaks were observed in that range either with or without the use of the argon neutralizer gun.

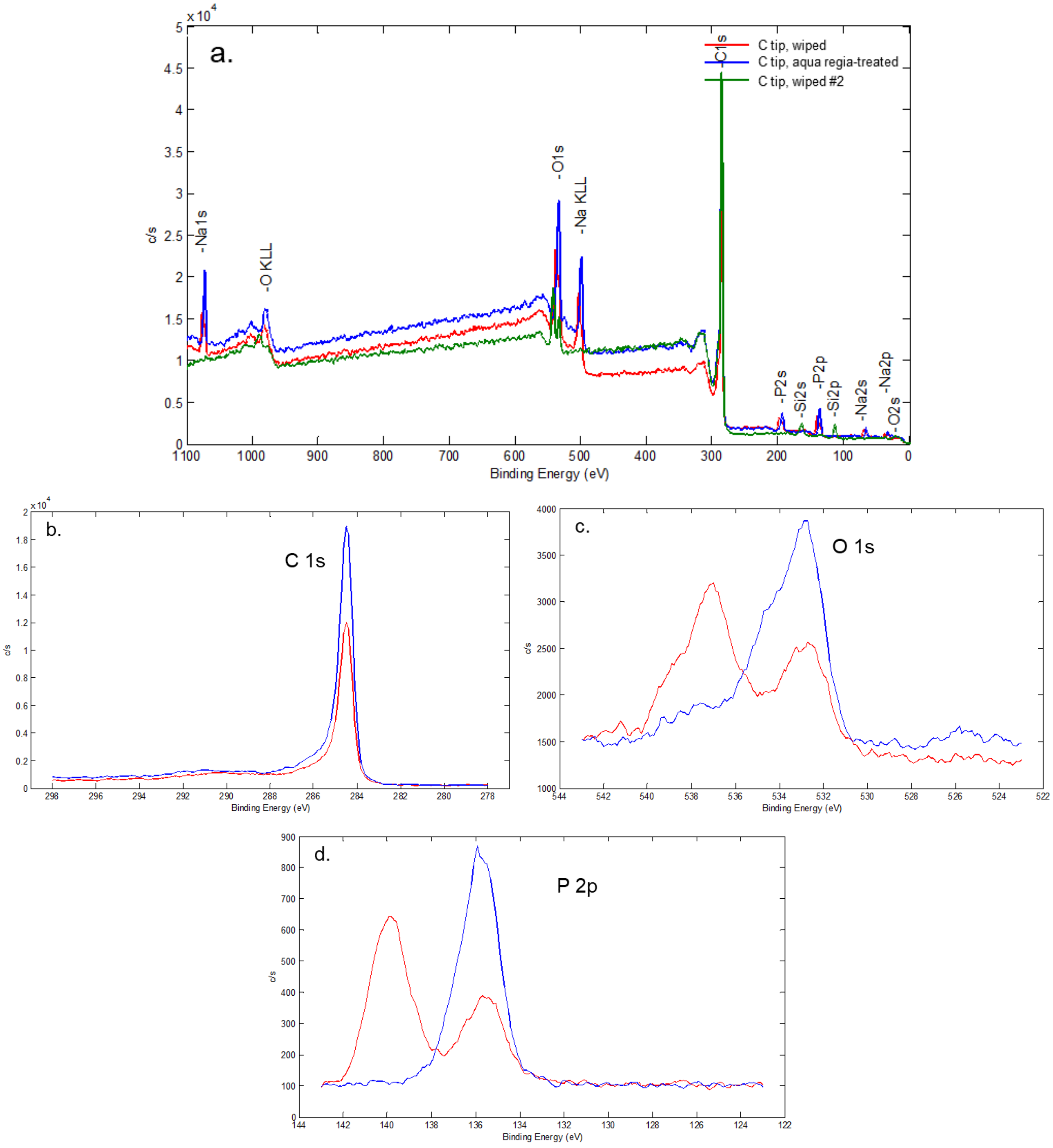

Figure S26. (*a*) Survey XPS spectrum of three graphite cathode tips following steady-state phosphorus evolution. (*b*) C 1s XPS spectrum of a graphite cathode tip. Red: no charge neutralization; blue: neutralization with argon beam. (*c*) O 1s XPS spectrum of a graphite cathode tip. Red: no charge neutralization; blue: neutralization with argon beam. (*d*) P 2p XPS spectrum of a graphite cathode tip. Red: no charge neutralization; blue: neutralization with argon beam.

Section S3 – Supplemental References

(*numbering continued from main text sources*)

53. Haupin, W. E. Electrochemistry of the Hall-Heroult process for aluminum smelting. *J. Chem. Educ.* **1983**, *60*, 279.

54. Prasad, S. Studies on the Hall-Heroult aluminum electrowinning process. *J. Braz. Chem. Soc.* **2000**, *11*, 245–251.

55. Frank, W. B.; Haupin, W. E.; Vogt, H.; Bruno, M.; Thonstad, J.; Dawless, R. K.; Kvande, H.; Taiwo, O. A. Aluminum. In *Ullmann's Encyclopedia of Industrial Chemistry*; Wiley-VCH: **2009**.

56. Campbell, F. H. Contributions to the chemistry of gold. *Trans. Faraday Soc.* **1907**, *3*, 103–113.

57. Cherevko, S.; Topalov, A. A.; Zeradjanin, A. R.; Katsounaros, I.; Mayrhofer, K. J. J. Gold dissolution: towards understanding of noble metal corrosion. *RSC Advances*. **2013**, *3*, 16516–16527.

58. Yoon, Y.; Yan, B.; Surendranath, Y. Suppressing Ion Transfer Enables Versatile Measurements of Electrochemical Surface Area for Intrinsic Activity Comparisons. *J. Am. Chem. Soc.* **2018**, *140*, 2397–2400.

59. Korzeniewski, C.; Conway, B. E. In *Proceedings of the Symposium on the Electrochemical Double Layer*; The Electrochemical Society: **1997**; vol. 97 of *Electrochemical Society Proceedings*; pp 349–350.

60. Chen, P.; Zhang, H.-B.; Lin, G.-D.; Hong, Q.; Tsai, K. R. Growth of carbon nanotubes by catalytic decomposition of CH_4 or CO on a Ni/MgO catalyst. *Carbon*. **1997**, *35*, 1495–1501.

61. Wiberg, E.; Holleman, A. F.; Wiberg N. In *Inorganic Chemistry*; Academic Press: **2001**; pp 810-811.

62. Basu, P. Gasification Theory. In *Biomass Gasification, Pyrolysis and Torrefaction*, 3rd ed.; P. Basu, Ed.; Academic Press: **2018**; pp 211-262.

63. Greenfield, S.; Clift, M. *Analytical Chemistry of the Condensed Phosphates*; Pergamon: Oxford, **1975**.

64. Feki, M.; Ayedi, H. F.; Purification of wet process phosphoric acid by solvent extraction with methyl isobutyl ketone: systematic study of impurity distribution. *Sep. Sci. Technol.* **2008**, *33*, 2609-2622.
